# PAX6 Regulates Melanogenesis in the Retinal Pigmented Epithelium through Feed-Forward Regulatory Interactions with MITF

**DOI:** 10.1371/journal.pgen.1004360

**Published:** 2014-05-29

**Authors:** Shaul Raviv, Kapil Bharti, Sigal Rencus-Lazar, Yamit Cohen-Tayar, Rachel Schyr, Naveh Evantal, Eran Meshorer, Alona Zilberberg, Maria Idelson, Benjamin Reubinoff, Rhonda Grebe, Rina Rosin-Arbesfeld, James Lauderdale, Gerard Lutty, Heinz Arnheiter, Ruth Ashery-Padan

**Affiliations:** 1 Department of Human Molecular Genetics and Biochemistry, Sackler Faculty of Medicine, Tel Aviv University, Tel Aviv, Israel; 2 Unit on Ocular and Stem Cell Translational Research, National Eye Institute, National Institutes of Health, Bethesda, Maryland, United States of America; 3 Department of Genetics, The Institute of Life Sciences, The Hebrew University of Jerusalem, Jerusalem, Israel; 4 Department of Clinical Microbiology and Immunology, Sackler School of Medicine, Tel Aviv University, Tel Aviv, Israel; 5 The Hadassah Human Embryonic Stem Cell Research Center, The Goldyne Savad Institute of Gene Therapy & Department of Gynecology, Hadassah-Hebrew University Medical Center, Jerusalem, Israel; 6 Wilmer Ophthalmological Institute, The Johns Hopkins University, School of Medicine, Baltimore, Maryland, United States of America; 7 Department of Cellular Biology, The University of Georgia, Athens, Georgia, United States of America; 8 Mammalian Development Section, National Institute of Neurological Disorders and Stroke, National Institute of Health, Bethesda, Maryland, United States of America; New York University, United States of America

## Abstract

During organogenesis, PAX6 is required for establishment of various progenitor subtypes within the central nervous system, eye and pancreas. PAX6 expression is maintained in a variety of cell types within each organ, although its role in each lineage and how it acquires cell-specific activity remain elusive. Herein, we aimed to determine the roles and the hierarchical organization of the PAX6-dependent gene regulatory network during the differentiation of the retinal pigmented epithelium (RPE). Somatic mutagenesis of *Pax6* in the differentiating RPE revealed that PAX6 functions in a feed-forward regulatory loop with MITF during onset of melanogenesis. PAX6 both controls the expression of an RPE isoform of *Mitf* and synergizes with MITF to activate expression of genes involved in pigment biogenesis. This study exemplifies how one kernel gene pivotal in organ formation accomplishes a lineage-specific role during terminal differentiation of a single lineage.

## Author Summary

It is currently poorly understood how a single developmental transcription regulator controls early specification as well as a broad range of highly specialized differentiation schemes. PAX6 is one of the most extensively investigated factors in central nervous system development, yet its role in execution of lineage-specific programs remains mostly elusive. Here, we directly investigated the involvement of PAX6 in the differentiation of one lineage, the retinal pigmented epithelium (RPE), a neuroectodermal-derived tissue that is essential for retinal development and function. We revealed that PAX6 accomplishes its role through a unique regulatory interaction with the transcription factor MITF, a master regulator of the pigmentation program. During the differentiation of the RPE, PAX6 regulates the expression of an RPE-specific isoform of *Mitf* and importantly, at the same time, PAX6 functions together with MITF to directly activate the expression of downstream genes required for pigment biogenesis. These findings provide comprehensive insight into the gene hierarchy that controls RPE development: from a kernel gene (a term referring to the upper-most gene in the gene regulatory network) that is broadly expressed during CNS development through a lineage-specific transcription factor that together with the kernel gene creates *cis*-regulatory input that contributes to transcriptionally activate a battery of terminal differentiation genes.

## Introduction

The retinal pigmented epithelium (RPE) is a monolayer of polarized and highly specialized pigmented cells that are located between the outer segments of the photoreceptors and the choroid layer in the eye. This strategic location demands multiple functions of the RPE during the development and homeostasis of the adjacent tissues, the neuroretina and choroid [1]. The RPE is a major component of the blood retinal barrier and it therefore determines the microenvironment of the photoreceptors. RPE cells are also responsible for photoreceptor outer segment phagocytosis and are directly involved in retinoid metabolism [1]. An important and evolutionarily conserved role of the RPE is the absorption of stray light to increase visual acuity and reduce oxidative damage. This latter activity requires functional melanosomes, which contain enzymes that catalyze the production of melanin (e.g. tyrosinase, TYR; tyrosinase-related protein, TYRP1; and dopachrome tautomerase, DCT) [2], [3]. Melanosomes accumulate in the RPE during cellular differentiation [4]. Defects in any of these complex functions of the RPE may lead to photoreceptor degradation and, eventually, blindness. Considering the importance of the RPE for ocular physiology and the recent breakthroughs in technologies involving gene transfer and cellular based therapies for treating RPE malfunctions, there is a need to understand the molecular and cellular mechanisms that regulate the acquisition of the various specialized functions of this important tissue.

Most pigmented cells in the body originate from the neural crest. In contrast, the RPE is derived from the neural epithelium of the optic vesicles (OV), which are lateral protrusions of the ventral forebrain. The OV undergo patterning and morphogenesis to give rise to the bilayer optic cup (OC) with an inner layer of retinal progenitor cells and an outer layer populated by the progenitors of the pigmented epithelium. The distal regions of the OC differentiate into the epithelial layers of the ciliary body and iris [5]. The partitioning of the optic neuroepithelium into neuronal and pigmented precursors depends on the activity of extrinsic and intrinsic cues such as transforming growth factor-betab (TGFb) and WNT ligands, which promote RPE development, and fibroblast growth factors (FGFs), which play a role in inducing neuronal fates [6]–[13].

Extrinsic cues trigger the expression and activity of intrinsic factors that execute the differentiation program. A pivotal intrinsic mediator of the RPE fate is microphthalmia-associated transcription factor (MITF), a member of the basic-helix-loop-helix leucine zipper family known to be essential for melanin-bearing pigment cells across species and tissue types [14]. MITF binds the DNA as a homodimer to stimulate the expression of its target genes [15], [16]. MITF is also able to form DNA-binding heterodimers with the related factors TFE3, TFEB and TFEC [17]. The *Mitf* gene encodes a family of at least 10 distinct isoforms generated from a common gene by alternative promoter/exon usage [18], [19]. Of these, *M*-*Mitf* is expressed in the neural crest derived pigment cells [20], [21], and *A-Mitf*, *H*-*Mitf* and *D*-*Mitf* are highly expressed in the developing RPE where they are equally distributed [18], [22]. The mRNAs of *Mitf* isoforms *M*, *A*, *H* and *D* contain different non-coding and coding 5′ sequences and the corresponding proteins thus differ in their N-terminal sequences [18], [22]. However, each of these protein isoforms can regulate the expression of the melanogenic genes [22]–[26].

In melanocytes, PAX3, SOX10, CREB and the canonical WNT3A signaling pathway regulate the expression of the *M-Mitf* isoform [27]–[31]. In contrast, in the RPE, where *Pax3* is not expressed, data suggest that PAX6 plays a role in regulating the onset of *Mitf* expression [32]. *Pax6*, the homolog of the *eyeless* (*ey)* gene in *Drosophila*, is pivotal for development of eye cell types derived from the neuroepithelium or from the surface ectoderm (reviewed in [33] and [34]). Moreover, ectopic expression of *Pax6* in frog embryos leads to formation of a differentiated eye, thus demonstrating a role for PAX6 in the different ocular lineages including the RPE [35]. The evolutionarily conserved roles of PAX6 and its upstream regulatory functions suggest that in ocular tissue types *Pax6* is a kernel gene, a term referring to the hierarchically upper-most gene in a gene regulatory network [36].

*Pax6* is abundantly expressed during patterning of the OV and during specification and differentiation of the RPE. Several studies indicate that PAX6 is important in the specification of the RPE. Recently it was shown that a reduction in *Pax6* gene dosage leads to development of neuroretina instead of RPE in embryos that are heterozygous for a mutation in *Mitf*, while embryos with such a mutation and normal *Pax6* levels do not exhibit any detectable phenotype [37]. Furthermore, at the OV stage, the redundant activities of PAX6 and PAX2 are required for the early patterning of the OV by regulating *Mitf* expression [32]. Later in development, PAX6, but not PAX2, is detected in the RPE [32]. These findings establish a role for PAX6 during the RPE specification stage and imply that PAX6 is also important during the differentiation of the RPE, although its role at this stage is still unknown.

The goal of this study was to examine the roles of *Pax6* during RPE differentiation, after the specification of the RPE is established. We show that during the onset of RPE differentiation PAX6 regulates the expression of *Mitf* and at the same time PAX6 functions together with MITF to activate the expression of downstream targets that execute melanogenesis in the RPE. Our findings reveal the molecular mechanism through which a single transcription factor, which is expressed in multiple ocular and non-ocular cell types, controls a highly specialized differentiation program of the neuroepithelium-derived pigmented cells of the eye.

## Results

### PAX6 is required for the pigmentation program of the RPE

**Figure 1:**
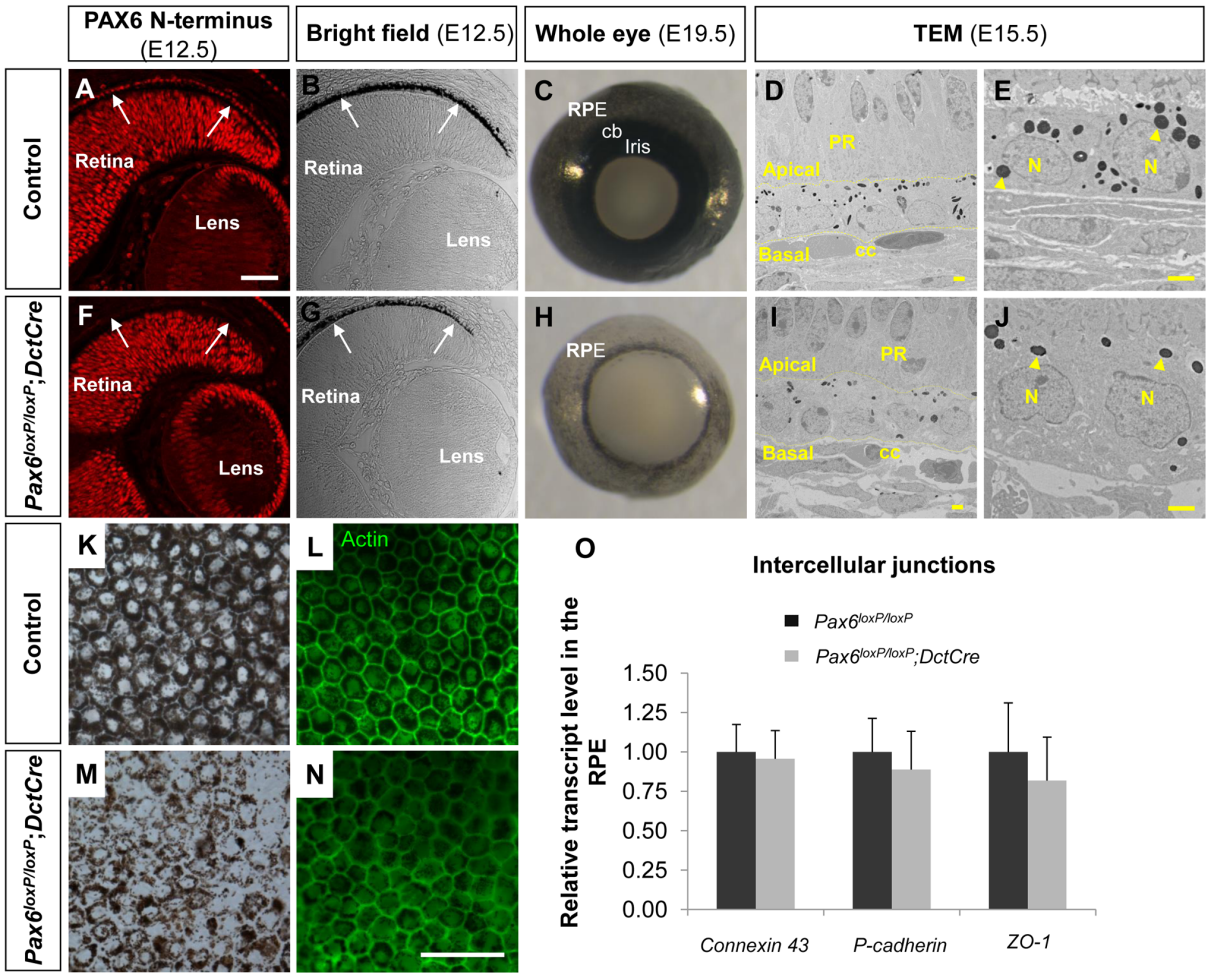
PAX6 expression is essential for proper pigment accumulation in the RPE but dispensable for RPE polygonal and single layer morphology. (A-N) RPE of (A-E,K,L) *Pax6^loxP/loxP^* and (F-J,M,N) *Pax6^loxP/loxP^;DctCre* mice analyzed for (A,F) PAX6 expression, (B,C,E,G,H,J,K,M) pigment accumulation and (D,L,I,N,O) morphology and specification. (A,F) Paraffin sections of E12.5 eyes were stained for PAX6 N-terminus and (B,G) viewed by differential interference contrast imaging. Scale bar is 100 µm. (C,H) Whole eye images of E19.5 mice. (D,E,I,J) Transmission electron microscope images of E15.5 eyes. Dashed lines mark the apical and basal membranes of the cells; arrowheads indicate melanosomes. Scale bar is 2 µm. (K-N) RPE flat-mount views of E19.5 eyes (K,M) using bright field or (L,N) stained for actin. Scale bar is 100 µm. (O) Relative transcript levels of *connexin-43* (a gap junction marker), *P-cadherin* (an adherens junction marker) and *ZO-1* (a tight junction marker) from control and *Pax6*-deficient E15.5 RPE fractions determined using QRT-PCR (n = 6). Abbreviations: CB, ciliary body; CC, choriocapilaris; N, nucleus; PR, photoreceptors.

Once the optic cup has formed (around E10.5), RPE progenitors begin to accumulate melanin [38], [39]. During the initiation of the pigmentation program, the expression of PAX6 is detected throughout the RPE layer (E10- E12.5, Figure 1A). In later stages, the expression of PAX6 is gradually reduced, first in the central and subsequently in the peripheral optic cup (Figure S1A-D). PAX6 is eventually maintained in the pigmented cells of the ciliary body (CB) and iris. To study the role of PAX6 in the RPE after its specification and during the first steps of its differentiation, we generated *Pax6^loxP/loxP^;DctCre* mice in which *loxP* sites are located in exon 4 upstream of the initiator ATG and in intron 6 [5], [40], [41]. The *Dct* promoter is active in the dorsal side of the OV at E9.5 and by E12.5 its activity is detected in the outer layer of the OC in RPE progenitors [5], [41].

Corresponding with *DctCre* activity and the location of the *loxP* sequences, the PAX6 paired domain was lost from the optic cup as evident from labeling with an antibody that specifically identifies the N-terminus of PAX6 (E12.5, Figure 1F and Figure S1A-H, red). Nevertheless, a C-terminal fragment of PAX6 was detected in the *Pax6^loxP/loxP^;DctCre* mice when using polyclonal antibodies that detect this region of the protein (Figure S1, green). The expression of this variant lacking the paired domain (PD) of PAX6 (PAX6ΔPD) was transient and reliably mimicked that of the full-length PAX6 during development as the PAX6ΔPD gradually disappeared in a central to peripheral pattern and was eventually lost from the RPE at around birth (Figure S1, green).

We further characterized the expression of *Pax6ΔPD* transcripts in the mutant RPE: a first *Pax6ΔPD* transcript variant was generated from the P4 promoter and was also detected in control RPE at E16 by RT-PCR and *in situ* hybridization using a specific probe (Figure S2A,C,E). A second *Pax6ΔPD* variant was generated due to aberrant splicing between exon 3 and 7 (Figure S2B,D). Nonetheless, we did not detect over-expression of exons 7-8, located upstream of the homeodomain (HD), in the mutated RPE by quantitative real-time PCR (QRT-PCR, Figure S2F). Thus, the *Pax6^loxP/loxP^;DctCre* mice constitute a genetic model for determining the role of the full-length PAX6 protein, while not excluding activities mediated by PAX6ΔPD.

The phenotype of the *Pax6^loxP/loxP^;DctCre* eyes was evident during embryogenesis as the iris and CB progenitors, which are evident at E19.5 (Figure 1C), did not develop in the *Pax6^loxP/loxP^;DctCre* OC (Figure 1H) in agreement with a previous report [5]. In addition, reduced pigmentation in the RPE was noted from early stages of RPE differentiation (E12.5, Figure 1B,G) and was evident when viewing the whole eye of *Pax6^loxP/loxP^;DctCre* as compared to control litter mates (E19.5, Figure 1C,H) or in flat mount (E19.5, Figure 1K,M).

Although pigmentation was reduced, the fate of the RPE was maintained in the *Pax6*-mutant RPE based on the expression of the transcription factors *Otx2* and *Sox9* (Figure S3). Consistent with maintenance of RPE fate, transmission electron microscope (TEM) analysis conducted on E15.5 control and *Pax6^loxP/loxP^;DctCre* eyes (Figure 1D,E,I,J) demonstrated that the typical RPE morphology of a single layer was preserved despite reduction in pigmentation. The adjacent structures of the choriocapillaris and neuroretina maintained normal morphology despite *Pax6* loss in the RPE (Figure 1D,I). We next examined actin distribution by phalloidin staining in flat mounts of the RPE and observed that the typical polygonal morphology of the RPE was maintained (Figure 1L,N). Moreover, using QRT-PCR analysis we did not detect significant differences between control and *Pax6*-deficient RPE in the levels of mRNAs encoding the intercellular junction proteins ZO-1, Connexin-43 and P-cadherin (E15.5, Figure 1O). These findings reveal a role for PAX6 in execution of the pigmentation program, although its absence does not alter the fate and morphology of the RPE at the OC stage.

### PAX6 is required for the expression of key melanogenic genes

To determine the global change in gene expression following *Pax6* loss in the OC we determined the transcript profile in control and *Pax6^loxP/loxP^;DctCre* E15.5 RPE using Affymetrix GeneChip Mouse Gene 1.0 ST arrays. Of the 28,853 genes represented on the microarray, levels of 100 transcripts were significantly altered in mutant RPE, compared to the wild-type (fold change greater than 1.5, *p*<0.05, Table S1). The expression of 73 of these genes was reduced in the *Pax6*-deficient RPE. In agreement with the observed phenotype, analysis of enrichment in GO categories revealed significant representation of melanogenic genes (*p*<0.05; using the ToppGene Suite algorithm; [42]) as summarized in Table 1. The identified pigmentation genes encode key enzymes of melanogenesis (*Tyr* and *Tyrp1*), as well as factors involved in melanosome biogenesis (*Si*, *Mlana*, and *Gpr143*) or melanosome transport (*RAB27a*) and factors implicated in melanosome biogenesis (*Gpnmb*, *Slc45a2*, *Slc24a5* and *Slc3a2*). Corresponding to the phenotype observed, the transcriptome analysis indicates an arrest in the melanogenesis program following *Pax6* loss.

**Table 1:** Melanogenic genes down-regulated in the *Pax6*-deficient RPE.

Gene symbol	Gene name	Fold change	*p* value	References
*Gpnmb*	Glycoprotein (transmembrane) nmb	−5.73	0.00020	[48], [87]
*Mlana*	Melan-A	−4.17	0.00018	[47]
*Gpr143*	G protein-coupled receptor 143	−2.83	0.00267	[88], [89]
*Tyrp1*	Tyrosinase-related protein 1	−1.91	0.00297	[46]
*Slc45a2*	Solute carrier family 45, member 2	−1.90	0.00630	[90], [91]
*Rab27a*	Member RAS oncogene family	−1.81	0.00530	[92]
*Slc24a5*	Solute carrier family 24, member 5	−1.68	0.03025	[93]
*Slc3a2*	Solute carrier family 3 (activators of dibasic and neutral amino acid transport), member 2	−1.66	0.00025	[94]
*Si*	Silver	−1.60	0.00721	[47], [95]
*Tyr*	Tyrosinase	−1.58	0.04166	[46]

**Figure 2:**
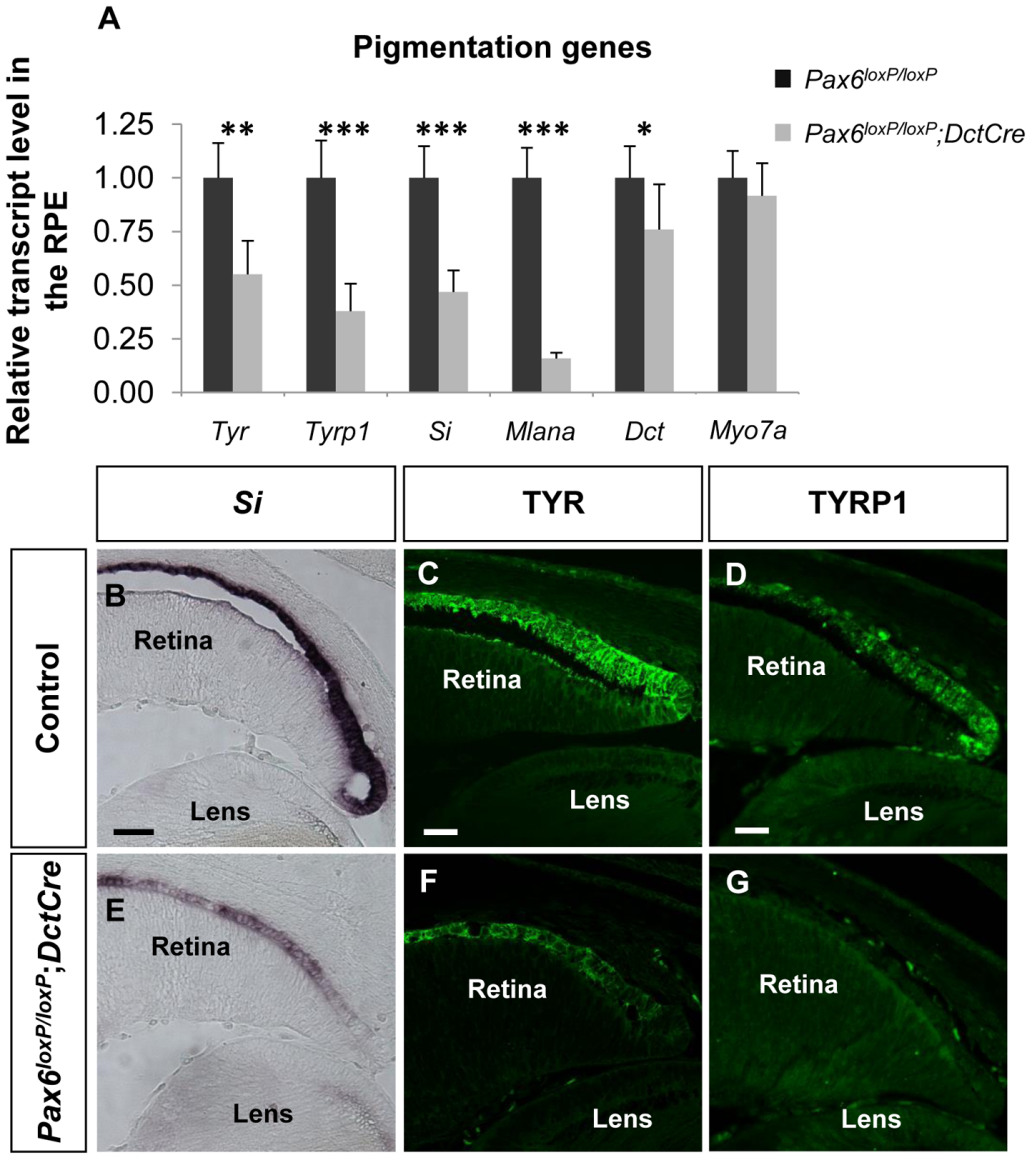
PAX6 is required for the expression of several melanogenesis genes. (A) Relative levels of *Tyr*, *Tyrp1*, *Si*, *Mlana*, *Dct* and *Myo7a* transcripts in RPE of control *Pax6^loxP/loxP^* and mutant *Pax6^loxP/loxP^;DctCre* E15.5 mice determined using QRT-PCR. *p<0.05, **p<0.005, ***p<0.0005, (n = 5). (B-G) Control and mutant RPE (B,E) cryo-sections showing the distal OC subjected to *in situ* hybridization for *Si* and (C,D,F,G) paraffin sections labeled with antibodies against TYR and TYRP1. Scale bar is 50 µm in B and E and 25 µm in C,D,F,G.

To validate the microarray results, six melanogenic genes were analyzed by QRT-PCR (Figure 2A). In agreement with the microarray results, transcript levels of *Tyr*, *Tyrp1*, *Si* and *Mlana* were significantly reduced in the mutant RPE as compared with control, whereas the level of the mRNA encoding the enzyme DCT, which is involved in melanin synthesis, was slightly reduced, and the level of *Myo7a* mRNA, which encodes a protein involved in cellular transport of melanosomes in the RPE [43], was similar to wild-type. The reductions of *Si* transcript (Figure 2B,E) and of TYR and TYRP1 proteins (Figure 2C,D,F,G) were validated by *in situ* hybridization and antibody labeling, respectively. These findings support a role for PAX6 in the proper expression of key melanogenic genes in the RPE.

### PAX6 is required for the expression of *D-Mitf* in the developing RPE

The transcription factor *Mitf* is considered the master regulator of all melanin-bearing pigment cells and several melanogenic genes are direct targets of MITF [44], [45]. Out of the 10 melanogenic genes found to be down-regulated following *Pax6* loss, seven are known direct targets of MITF: *Tyr* and *Tyrp1* [46], *Si* and *Mlana* [47], *Gpnmb* [48], *Rab27a* [49] and *Gpr143* [50]. *Mitf* has previously been found to be regulated by PAX6 and PAX2 at the OV stage [32]. We therefore wanted to investigate whether *Mitf* expression is dependent on PAX6 after RPE specification, when PAX2 is not expressed in the pigmented epithelium [32].

**Figure 3:**
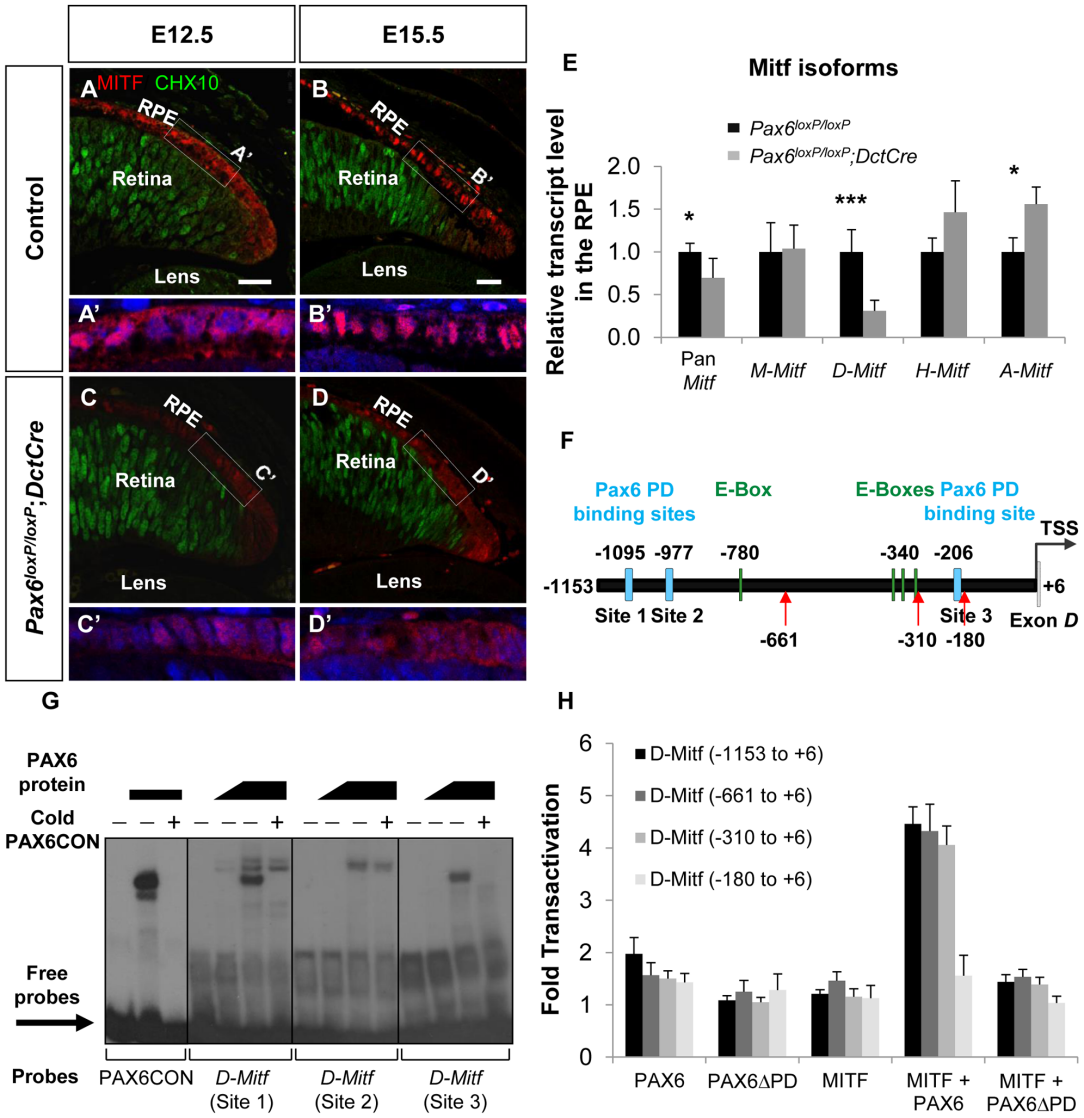
PAX6 is required for the expression of the *D*-*Mitf* isoform in the developing RPE. (A-D) Expression of MITF (red) and CHX10 (green) proteins detected by antibody labeling in the RPE of *Pax6^loxP/loxP^* control and *Pax6^loxP/loxP^;DctCre* mutant E12.5 and E15.5 eyes. Scale bar is 25 µm. (A'-D' insets) Higher magnifications of indicated regions and nuclear staining with DAPI. (E) Relative transcript levels of pan*-Mitf* and *M-*, *D-*, *H-* and *A-Mitf* isoforms in RPE fractions using QRT-PCR, *p<0.05, ***p<0.0005, (n = 5). (F) A scheme of the *D-Mitf* upstream region showing the putative E-boxes (green rectangles) and PAX6 PD binding sites (light blue rectangles). Red arrows indicate the borders of deletion constructs used for luciferase assay. (G) EMSA examining the binding of PAX6 to the putative PAX6 PD binding sites upstream of the *D-Mitf* TSS (sites 1-3). The binding of PAX6 to probes 1 and 3 was inhibited using unlabeled probe containing the PAX6 consensus binding site (PAX6CON). (H) Activity of luciferase under the regulation of wild-type or truncated *D-Mitf* promoter co-transfected into HeLa cells along with different combinations of expression vectors and/or their backbones lacking the ORF (n = 3).

The *Mitf* gene encodes a family of isoforms generated from a common gene. The isoforms that are predominantly expressed in the RPE are *A*, *H* and *D* [18] (Figure S4A,B). The average response of all the *Mitf* probes in the GeneChip array revealed a reduction in the transcript levels by 1.37 fold (*p* = 0.069) in *Pax6*-deficient RPE compared to the wild-type. By indirect immunofluorescence (IIF) analysis we indeed detected reduced levels of MITF protein in the RPE of *Pax6^loxP/loxP^;DctCre* embryos as compared with control *Pax6^loxP/loxP^* embryos at E12.5 and at E15.5 (Figure 3A-D). We next determined the expression levels of the specific *Mitf* isoforms by QRT-PCR of RNA extracted from control and *Pax6*-deficient RPE (E15.5). This analysis revealed a significant reduction in the expression of *D*-*Mitf*, which was over 3-fold lower in the mutants, and slight elevations in levels of *A*-*Mitf* and *H*-*Mitf* in the mutant RPE, compared to wild-type (Figure 3E). Quantification of a downstream amplicon that is common to all *Mitf* isoforms revealed a significant 1.45-fold reduction in pan-*Mitf* transcripts, consistent with the microarray results. *In silico* analysis of the upstream regulatory region of *D-Mitf* revealed three putative binding sites for PAX6 PD and four for MITF (Table S2); all are located within the 1200bp preceding the *D-Mitf* transcription start site (TSS) (Figure 3F). An electrophoretic mobility shift assay (EMSA) revealed that PAX6 binds two of the three sites *in vitro* (Figure 3G). This binding was specific, as it was competed by a cold probe (Figure 3G). Luciferase reporter assay on regulatory sequences of *D*-*Mitf* (between −1,153 and +6 relative to the TSS) was performed using different combinations of *Pax6*, *Pax6ΔPD* and *Mitf* expression vectors (Figure 3H). This analysis revealed synergistic transactivation of the *D-Mitf* promoter by PAX6 and MITF. The co-transfection of MITF with PAX6ΔPD failed to produce the same result.

In order to identify the regulatory sequence required for PAX6 and MITF transcriptional activity, a series of *D-Mitf* truncated promoters was analyzed (Figure 3H). The critical regulatory sequence for PAX6 and MITF transactivation is located between −310 and −180 relative to the TSS. This region contains only a PAX6 binding site (site 3: −212 and −194 relative to the TSS) but no known MITF binding site. Taken together, these results suggest that the transactivation of *D-Mitf* promoter by PAX6 and MITF depends on PAX6 PD and requires the PD binding site. These results also indicate that a self-sustaining PAX6-dependent feedback loop controls *Mitf* expression.

### *D-Mitf* is dispensable for melanogenesis in the RPE

**Figure 4:**
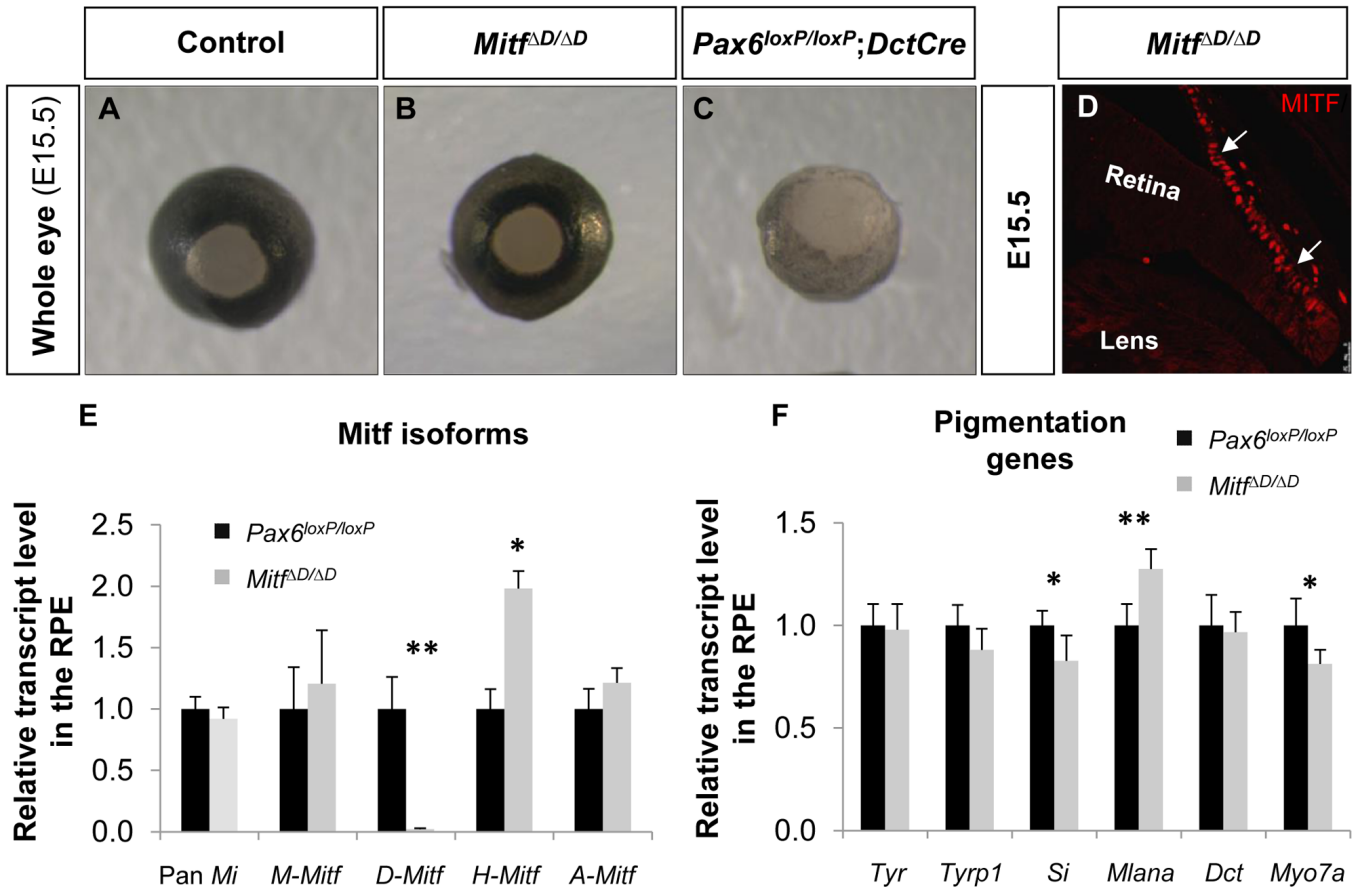
*D-Mitf* is dispensable for melanogenesis in the RPE. (A-C) Whole eye images of (A) *Pax6^loxP/loxP^*, (B) *Mitf^ΔD/ΔD^* and (C) *Pax6^loxP/loxP^;DctCre* mice. (D) A distal OC view of paraffin section of a *Mitf^ΔD/ΔD^* eye labeled with antibody against MITF. Arrows point at the RPE. (E) Relative transcript levels of pan*-Mitf* and *M-*, *D-*, *A-* and *H-Mitf* isoforms in RPE fractions determined using QRT-PCR. (F) Relative transcript levels of *Tyr*, *Tyrp1*, *Si*, *Mlana*, *Dct* and *Myo7a* in RPE fractions determined using QRT-PCR. *p<0.05, **p<0.005, (n = 5).

The abrogated melanogensis in the *Pax6*-deficient RPE and the corresponding reduction in the *D*-*Mitf* isoform suggested that the pigment depletion in the *Pax6*-deficient RPE could be mediated by *D-Mitf*. Recently mice with specific deletion of *D*-*Mitf* were generated by ablation of 0.2 kb downstream to exon *D*, exon *D* and the 5.6 kb preceding sequence (*Mitf^ΔD^*, Figure S4C). In these mice, a slight reduction in pigmentation was observed at E11; however, at later stages the pigmentation was comparable to normal, in contrast to the depigmentation observed in the *Pax6^loxP/loxP^;DctCre* mutant eyes [37] (Figure 4A-C). Molecular analysis of *Mitf^ΔD/ΔD^* by IIF and QRT-PCR revealed that pan-*Mitf* level was similar to that in wild-type mice (Figure 4D,E). Yet, the expression levels of *Mitf* variants in the *Mitf^ΔD/ΔD^* RPE were similar to the pattern observed in the *Pax6^loxP/loxP^;DctCre* mutants: *D*-*Mitf* transcript level was completely abolished while *A*-*Mitf* and *H*-*Mitf* expression levels were elevated (Figure 4E). In addition, transcript quantification of the six melanogenic genes examined in the *Pax6*-deficient mutants revealed minor reductions in the levels of *Tyrp1*, *Si* and *Myo7a*, but only the reductions of the latter two were significant (Figure 4F). The normal phenotype of *Mitf^ΔD/ΔD^* mice is probably due to redundant activity of the *Mitf* isoforms expressed in the RPE. Together, the above results reveal that while PAX6 is required for normal levels of expression of *D-Mitf*, the reduced levels of *D*-*Mitf* following *Pax6* loss cannot account for the observed arrest in melanogenesis in the *Pax6^loxP/loxP^;DctCre* mutants. Moreover, the dramatic loss of pigmentation, while levels of *Mitf* are partly maintained, indicates that PAX6 has other functions in melanogenesis of the RPE in addition to the regulation of *Mitf* levels.

### PAX6 enhances the transcriptional activity of MITF on downstream melanogenic factors

**Figure 5:**
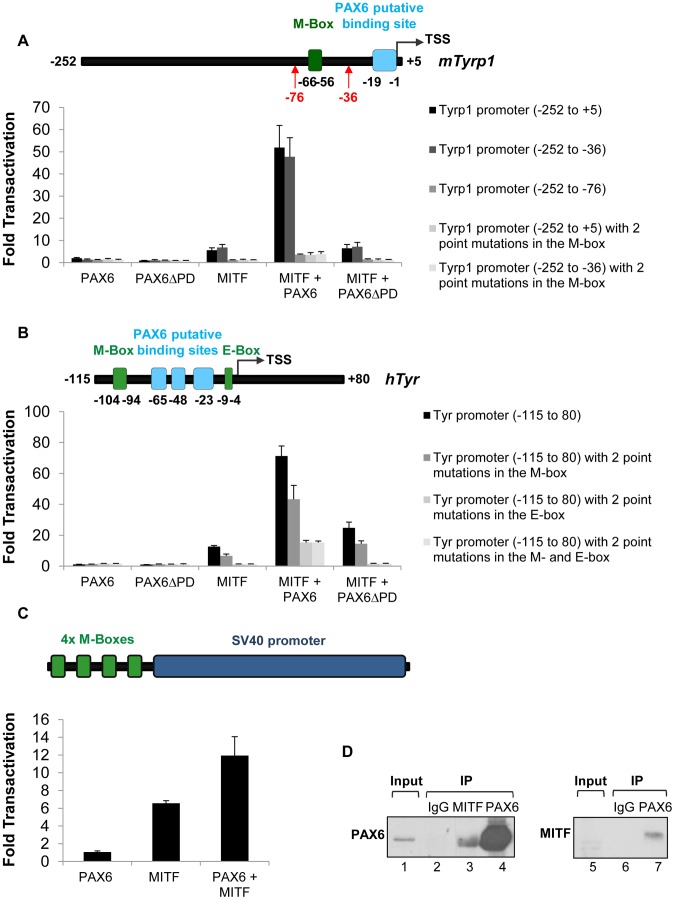
PAX6 trans-activates the promoters of *mTyrp1* and *hTyr* in the presence of MITF. (A,B) Activity of luciferase under the regulation of wild-type or mutated (A) *mTyrp1* or (B) *hTyr* promoters co-transfected into HeLa cells along with different combinations of expression vectors and/or their backbones lacking the ORF, as indicated (n = 3). The positions of binding sites for MITF (E/M-box, green rectangle) and potential binding sites for PAX6 (light blue rectangle) are indicated relative to the TSS of each promoter in schematics above each graph. (C) Activity of luciferase under the regulation of four consecutive M-boxes and a basic SV40 promoter co-transfected into HeLa cells along with different combinations of expression vectors and/or their backbones lacking the ORF, as indicated (n = 3). (D) Reciprocal co-immunoprecipitation assay of PAX6 and MITF using protein extracts of ARPE19 cells. Samples were precipitated using anti-PAX6 (lanes 4,7), anti-MITF (lane 3) or IgG (lanes 2,6). Anti-Pax6 (lanes 1-4) or anti-MITF (lanes 5-7) were used for Western blot.

The findings above reveal that PAX6 plays a pivotal role in the pigmentation program that goes beyond regulation of *D-Mitf* expression. This is reminiscent of the activity of the *Pax3* gene in melanocyte precursors, where it regulates the onset of *Mitf* expression as well as the expression of *Mitf* target genes like *Tyrp1* [51]. In order to examine the ability of PAX6 to trans-activate known targets of MITF we performed luciferase reporter assays using the regulatory regions of three pigmentation genes: *mTyrp1, hTyr* and *mMlana* (see Tables S3-S5 for details on MITF and PAX6 putative binding sites). We also examined the transcriptional activity of PAX6ΔPD, which was detected in *Pax6^loxP/loxP^;DctCre* mice (Figure S1 and Figure S2), on these promoters. On the *mMlana* promoter there was additive cooperation between the two transcription factors (PAX6: 2.25 fold change, *p* = 0.02; MITF: 50.3 fold change, *p* = 0.004; MITF + PAX6: 149 fold change, *p*≤0.04; n = 3; Figure S5). In contrast to PAX3, PAX6 by itself failed to activate the *mTyrp1* promoter either in pigment producing cell lines such as ARPE19 and UACC.62, or in HEK-293T, NIH-3T3 and HeLa cells (Figure 5A and data not shown). However, in the presence of MITF, PAX6 cooperatively and synergistically trans-activated the m*Tyrp1* promoter (MITF: 5.6 fold change, *p* = 0.006; MITF + PAX6: 51.9 fold change, *p* = 0.002; n = 4; Figure 5A). A similar synergistic transactivation pattern was observed using the *hTyr* promoter (MITF: 12.6 fold change, *p* = 0.001; MITF + PAX6: 71.3 fold change, *p* = 0.003; n = 3; Figure 5B). Chromatin immunoprecipitation (ChIP) confirmed the association of PAX6 with the *hTyrp1* promoter region in RPE cells derived from human embryonic stem cells [52]. We observed more than 4-fold enrichment of PAX6 in the *hTyrp1* proximal promoter compared to a region 2 kb downstream (data not shown and Table S6).

We next examined the contributions of the putative binding sites of PAX6 and the binding sites of MITF (M- and E-box) to the transactivation of the promoters of *mTyrp1* and *hTyr* by PAX6 and MITF. Interestingly, deletion or point mutations in the MITF binding sites dramatically reduced the transactivation observed when MITF and PAX6 were co-expressed. While the wild-type *mTyrp1* promoter was trans-activated 51.9 fold in the presence of both factors, compared to their absence, a promoter carrying a deletion or mutations in the M-box was trans-activated only 3.5 fold (Figure 5A). Similarly, the wild-type *hTyr* promoter was trans-activated 71.3 fold by both PAX6 and MITF, whereas promoters carrying mutations in the M- and E-box sequences were trans-activated 43.3 fold and 13.3 fold, respectively (Figure 5B). In contrast, deletion of the putative binding site for PAX6 in *mTyrp1* promoter did not significantly alter the transactivation by MITF and PAX6 (Figure 5A). To examine whether the M-box is sufficient to enhance MITF activity by PAX6, we performed a luciferase reporter assay with PAX6, MITF or both using a reporter with four consecutive M-box elements. As shown in Figure 5C, PAX6 alone did not activate the promoter, MITF alone enhanced the activity by 6.6 fold, and PAX6 and MITF together enhanced the reporter activity by 11.9 fold (*p* = 0.046, n = 3). These findings suggest that in tissue culture, the MITF binding sites are essential and sufficient for the transactivation of *mTyrp1*and *hTyr* promoters by PAX6 and MITF.

The reporter assays revealed that PAX6 transactivation effects are largely dependent on MITF expression and on its binding sites. This mode of action suggests a physical interaction between PAX6 and MITF. We therefore conducted co-immunoprecipitation assays (co-IP) to evaluate this possibility. First, a reciprocal co-IP experiment was performed in ARPE19 cells that endogenously express both *Pax6* and *Mitf* [53]. MITF antibodies co-precipitated PAX6, and immunoprecipitation with PAX6 antibodies resulted in precipitation of MITF (Figure 5D). The enrichment of MITF in the PAX6 immunoprecipitate was very significant as MITF expression was almost below detection in the input sample (Figure 5D, right panel, lane 5). These results support an association of MITF and PAX6 in ARPE19 cells.

To determine whether the PAX6ΔPD variant is capable of physical association with MITF, HeLa cells were transfected with 3xFLAG-PAX6, 3xFLAG-PAX6ΔPD, 3xFLAG-MITF or a combination of 3xFLAG-MITF with each PAX6 protein variant (Figure S6A). Cells were harvested and protein extracts were precipitated using MITF antibodies. Both PAX6 and PAX6ΔPD proteins were enriched in the immunoprecipitates when co-transfected with MITF (Figure S6A, right panel, lanes 9 and 10). These results suggest that the PAX6ΔPD variant is capable of associating with MITF as previously suggested [54]. We next conducted luciferase reporter assays using MITF and PAX6ΔPD. PAX6ΔPD had no transactivation effects on the transcriptional activity of MITF (Figure 5A,B) and did not show a dominant negative effect on the transactivation of the *mTyrp1* promoter by MITF and PAX6 (Figure S6B). These results indicate that although the PAX6ΔPD variant is capable of association with MITF, the PD domain is necessary for the PAX6-MITF-mediated transcriptional activation of melanogenic genes.

## Discussion

**Figure 6:**
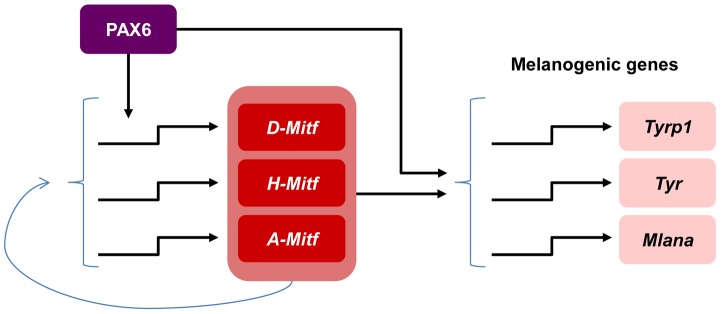
Model of PAX6 control of melanogenesis in the RPE through a positive feed forward loop with MITF. PAX6 positively regulates the expression of the *D*-*Mitf* isoform. There is a compensation mechanism that maintains pan-*Mitf* levels in the RPE. PAX6 cooperates with MITF to trans-activate several pigmentation genes.

This study unravels the molecular mechanism through which a single transcription factor, which is expressed in multiple ocular and non-ocular cell types, controls a highly specialized differentiation program of the neuroepithelium-derived pigmented cells of the eye. We show that PAX6 regulates a gene regulatory network central to RPE differentiation. This activity is mediated by a coherent feed-forward loop, by which PAX6 controls the expression of *Mitf* and jointly with MITF triggers the expression of multiple downstream target genes that are required for the execution of distinct differentiation program of pigment formation (Figure 6). In this mode of action, MITF levels could serve as a sign-sensitive delay for the melanogenesis process in the RPE as transactivation of pigmentation genes by PAX6 depends on *Mitf* transactivation by PAX6. This type of kinetic mechanism filters out fluctuations in input stimuli since it requires persistent co-expression of both transcription factors. Our data provide an explanation of how PAX6, which is expressed in most ocular lineages, can promote the highly specialized and distinct differentiation program of the RPE.

A role for *Pax6* during specification of the OV to the PE lineage was deduced from the analyses of *Pax6* mutants that also carry mutations in the transcription factors *Pax2* or *D-Mitf*. The PE of the transgenic *Pax2^−/−^;Pax6^+/−^* and *Mitf^ΔD/ΔD^;Pax6^+/−^* mice develops into a second neuroretina [32], [37]. In contrast, RPE cells that lost the expression of *Pax6* after specification maintained their morphology of a single layer of polygonal epithelium (Figure 1). Accordingly, we did not detect changes in the expression of several epithelial markers (Figure 1O) or elevated expression of the neuronal gene CHX10 (Figure 3A-D) in the *Pax6*-mutant RPE. Although the mutant RPE transcriptome data did not reveal overt elevations in neuronal genes, we did detect alterations in the expression levels of MITF-regulators, both key RPE-specification factors such as *Wnt2b* ([6]; −1.67, *p* = 0.00005) and *Gli2* ([55]; −1.1, *p* = 0.003) and of the retinal promoting gene *Msx2* ([56]; +1.8, *p* = 0.016). These alterations in gene expression suggest partial changes in the differentiation program of the *Pax6*-deficient RPE and point to additional regulators of MITF that are controlled by PAX6. However, these changes were not sufficient to completely disrupt RPE differentiation, in contrast to the complete disruption observed following inhibition of the Wnt/β-catenin pathway in specified RPE [57]. A previous study showed that PAX6 together with PAX2 is required for expression of MITF during the specification stage, and that the former two proteins regulate the expression of the *A-Mitf* isoform *in vitro* [32]. In our analysis, the loss of *Pax6* during RPE differentiation resulted in up-regulation of *A-Mitf*. These findings suggest stage-dependent roles for PAX6 during various stages of RPE development, from pattering to differentiation.

We show that *Pax6* is essential for the proper expression of *Mitf* and its melanogenic target genes. This activity requires both PAX6 and MITF to act synergistically, as shown by luciferase reporter assays on the promoters of *mD-Mitf*, *mTyrp1* and *hTyr* (Figure 3H and Figure 5A,B). Although PAX6 binding sites were identified in each of these three promoters (Tables S2-S4), the deletion of the PAX6 binding site in the *Tyrp1* promoter did not reduce the transcriptional activity of PAX6 and MITF, while mutations in MITF binding sites in either *mTyrp1* or *hTyr* promoters hampered their activity (Figure 5A,B). In contrast, the PAX6 binding site in the *D-Mitf* promoter (site 3, Figure 3F,G), but not the putative MITF binding sites, was essential for PAX6 and MITF transcriptional activity (Figure 3H). Together with the observation that PAX6 and MITF are capable of physical association (Figure 5D), these results suggest that the PAX6-MITF complex may trans-activate promoters through either PAX6 or MITF binding sites. This mode of action of PAX6 may account for the broad spectrum of PAX6 transcriptional targets. ChIP-Seq studies on embryonic RPE or hES-RPE cells are underway to determine the promoter occupancy of PAX6 and MITF on RPE genes.

The *Mitf* gene encodes at least 10 isoforms with alternative promoter or exon usage. The three RPE-specific isoforms (*D-*, *A-* and *H*-*Mitf*; [18], [19]) differ only in the N-terminal sequences [22]. The fact that all *Mitf* isoforms have different promoter sequences and predicted transcription factor binding sites suggests different regulation mechanisms [58]. Our data show that PAX6 is specifically required for the normal expression of *D-Mitf*. In both mouse mutant lines – *Pax6^loxP/loxP^;DctCre* and *Mitf ^ΔD/ΔD^* –we observed an up-regulation of *A*-*Mitf* and *H*-*Mitf* isoforms (Figure 3E and Figure 4E). However, while in the RPE of *Mitf^ΔD/ΔD^* mice the total transcript level of *Mitf* was similar to that in the wild-type RPE, in the RPE of *Pax6^loxP/loxP^;DctCre* the level of pan-*Mitf* was 1.45-fold lower than the wild-type RPE. We therefore suggest that there is a feedback mechanism that balances the total level of MITF protein and that this mechanism requires full-length PAX6.

The observation that *A*- and *H*-*Mitf* are capable of compensating for the absence of *D*-*Mitf* activity in the *Mitf^ΔD/ΔD^* transgenic mice, but not in the *Pax6^loxP/loxP^;DctCre* mutants, suggests that reduction in pigmentation in the RPE of *Pax6^loxP/loxP^;DctCre* transgenic mice might be caused by down-regulation of a MITF co-factor, either PAX6 itself or another protein. Other than MITF, the only transcription factor that has been demonstrated to have a role in regulation of RPE melanogenesis is OTX2. OTX2 plays an important role in RPE development [59], [60] by trans-activating the melanogenic enzymes-encoding genes *Tyr*, *Tyrp1* and *Dct* [61]–[63]. Since we did not detect a significant change in the expression pattern of *Otx2* in the RPE of *Pax6^loxP/loxP^;DctCre* transgenic mice (Figure S3), it is unlikely that changes in its expression mediate the reduction in the expression of the melanogenic genes and pigmentation observed following *Pax6* loss.

Another candidate that might be responsible for the reduced pigmentation in the *Pax6^loxP/loxP^;DctCre* is the bHLH leucine-zipper transcription factor TFEC. The amino acid sequences of TFEC and MITF bHLH leucine-zipper show high similarity [64] and these two proteins bind to an E-box as heterodimer complex [17]. Bharti et al. (2012) showed that PAX6 trans-activates the expression of *Tfec* and that *Tfec* can rescue eye defects in mice with a mutation in the *Mitf* gene [37]. The transcript level of *Tfec* was indeed reduced in *Pax6^loxP/loxP^;DctCre* mutants (Figure S7A: −1.23 fold change, *p* = 0.075, n = 6), and this minor down-regulation may have also contributed to the overall reduction in pigmentation, as TFEC is capable of trans-activating *mTyrp1* and *hTyr* promoters alone and synergistically with PAX6 (Figure S7B and data not shown). Thus, in addition to its known role during PE specification, TFEC may also have a role in RPE differentiation where it acts like an additional isoform of MITF [17].

Association between PAX6 and MITF was previously shown *in vitro* by Planque et al. (2001). However, in that study transfection of the two proteins caused a reduction in MITF transactivation of *Tyr* promoter [54]. The discrepancy with our results could be explained by the different ratio of *Pax6*/*Mitf* levels used in the reporter assays, as Planque et al. used a ratio of 20∶1, whereas in our study the ratio was 1∶1. The importance of the ratio between PAX6 and MITF has also been demonstrated *in vivo*. The transcript levels of *Tyr* and *Tyrp1* were lower in mice that over-express *Pax6* (i.e. *Pax6^Yac/Yac^* mice) on the background of a *Mitf^ΔD/ΔD^* transgenic mice compared to either *Pax6^Yac/Yac^* or *Mitf^ΔD/ΔD^* alone [37]. In these experiments, RPE pigmentation levels were consistent with the altered expression levels of *Tyr* and *Tyrp1* ([37] and Bharti, unpublished results). Interestingly, while during embryonic development PAX6 is eliminated from the RPE in a proximal to distal gradient, MITF and its downstream pigmentation genes are expressed along the entire length of the RPE. Thus, we infer that PAX6 is involved in the initiation of the pigmentation program but not in its maintenance.

The somatic mutation induced by the *DctCre* transgene deleted exons 4−6, which encode the initiation codon and the PD of PAX6. Interestingly, while the PD was eliminated from the *Pax6^loxP/loxP^;DctCre* embryos, a truncated transcript of *Pax6* that gave rise to a *Pax6ΔPD* variant was identified. The PAX6*Δ*PD variant was not previously noted in somatic mutations of the *Pax6^loxP^* allele [40], , probably because in some tissues, such as the lens placode and the peripheral optic cup, but not in the RPE, the expression of *Pax6* depends on full-length PAX6 protein [70], which is absent due to the Cre-mediated deletion. In addition, it is possible that RPE-specific post-transcriptional mechanisms that alter splicing and RNA stability lead to more prominent accumulation of the PAX6ΔPD in the RPE.

While the physiological activity of PAX6ΔPD in the eye is still unknown, its over-expression results in microphthalmia due to aberrant lens and corneal development [71], [72]. Thus, although we did not detect over-expression of the homeodomain of *Pax6*, we should consider the possible contribution of the over-expression of the PAX6*Δ*PD isoform and the disruption of the PAX6/PAX6*Δ*PD ratio to the pigment phenotype of the *Pax6^loxP/loxP^;Dct-Cre* RPE. There are several lines of evidence that rule out a major effect of the PAX6ΔPD isoform in the *Pax6^loxP/loxP^;Dct-Cre* mutants: First, we detected a *Pax6ΔPD* transcript in the control RPE, which was initiated from the P4 promoter (Figure S2A,C,E). Thus, the *Pax6ΔPD* transcript is expressed during normal differentiation and onset of pigmentation in the RPE. Second, *Pax6ΔPD* is expressed in the progenitors of the CB and is maintained there in the adult, both in the pigmented and non-pigmented epithelium [72]. Yet, mice carrying 10 copies of the *Pax6* locus and over-expressing *Pax6ΔPD* do not display any alteration in the pigmentation of the CB [72]. Therefore the PAX6ΔPD isoform is unlikely to interfere with the pigmentation program. Third, we did not detect reduced pigmentation in the RPE of *Pax6^loxP/+^;DctCre* heterozygous mice, thus further arguing against a dominant-negative effect of this truncated product ([5] and data not shown). Finally, even though the PAX6ΔPD isoform was able to associate with MITF in a co-IP assay (Figure S6A), it had no repressive or inductive effects on the promoters of *mTyrp1* and *hTyr* either alone or when co-expressed with MITF or together with MITF and PAX6 (Figure 5A,B and Figure S6B).

Although there is little evidence for independent eye invention events during metazoan species evolution [34], there is strong argument in favor of a common molecular network controlling the development of the metazoan eye, in which *Pax* genes were redundantly employed and were later on variably adapted for eye development in different animal taxa [34], . According to this hypothesis an ancestor of a *Pax6* gene was at the node of a gene regulatory network that controlled the morphogenesis of a primitive eye composed of a photoreceptor cell that contained pigment granules, as in *Palaemonetes pugio* [76]. The evolutionarily earliest gene regulatory networks were likely to be hierarchically shallow and, as animal body parts gradually elaborated and gained more complex regional subdivision of the developing embryo, the underlying regulatory networks became hierarchically deeper and were terminally fixed into kernel genes, in which any minor change would lead to extremely harmful consequences [36], [77]. In such a scenario, the development of the vertebrate eye into a complex structure that includes PE and multilayered neuroretina would require different cell specific transcription factors that in combination with PAX6 generate different *cis*-regulatory input functions that result in execution of distinct and highly specified differentiation programs. In this model, PAX6 acts as an accelerator directed by its tissue-specific partner to a specific transcriptional program.

## Material and Methods

### Mouse lines

The mouse lines employed in this study, *Mitf^ΔD^* [37], *Pax6^loxP^* [40] and *DctCre* [5] have been previously described. The latter two were used to establish *Pax6^loxP/loxP^;DctCre* somatic mutants. *Pax6^loxP/loxP^* littermates were used as controls. The genetic background of all mice used in this study was C57BL/6J, except for *in situ* staining, for which mice of the outbred ICR genetic background were used. All animal work was conducted according to national and international guidelines and approved by the Tel Aviv University review board.

### Statistical analysis

All data were examined using two-tailed Student's t-test.

### Immunofluorescence, ISH and flat-mount

Immunofluorescence analysis was performed on 10 µm paraffin sections as previously described [40], using the following primary antibodies: rabbit anti-PAX6 (1∶400, Covance, prb-278b), mouse anti-PAX6 (1∶25, Santa Cruz, sc-32766), rabbit anti-SOX9 (1∶200, Chemicon, ab5535), sheep anti-CHX10 (1∶1000, Exalpha, X1180P), rabbit anti-OTX2 (1∶50, Millipore, AB9566), rabbit anti-MITF [18], rabbit anti-TYR (1∶1,000, a gift from the Vincent Hearing lab, NCI), rabbit anti-TYRP1 (1∶1,000, a gift from the Vincent Hearing lab, NCI). Secondary antibodies were donkey anti-rabbit conjugated to alexa594 (1∶1000, Invitrogen, A21207) and alexa488 donkey anti mouse/sheep (1∶1000, Invitrogen, A21202/A11015).

*In situ* hybridization (ISH) was performed on 14 µm cryo-sections using DIG-labeled RNA probes as previously described [78]. The *Pax6* intron 7 probe was generated from a 849bp PCR fragment (forward, 5′-TTTGGAGCCCTCCATCTTTCTC-3′; reverse, 5′- TGCACACTTTCGGGCAAGG-3′). Plasmid for antisense transcription of *silver* was kindly provided by the laboratory of Dr. William Pavan (NIH) [79].

Flat-mount samples were prepared as follows: Eyes at E19.5 were enucleated and immediately fixed in 4% paraformaldehyde for 30 minutes. The RPE was carefully dissected from the rest of eye structures, sliced radially to four pieces and flattened on membrane filters (Schleicher& Schull, 0.45 µm D-37582). Samples were blocked and stained with phalloidin (1∶100, Invitrogen, A12379). Thereafter, RPE was flattened on its basal side on a slide and sealed for observation.

### Transmission electron microscopy

The heads of E15.5 embryos and perforated eyes of P1 neonatal mice were fixed in 0.1 M cacodylate-buffered fixative containing 2.5% paraformaldehyde and 2% glutaraldehyde and further processed as described previously [80]. Ultrathin sections were cut with a Leica Ultramicrotome UCT (Leica Microsystems), stained with uranyl acetate and lead citrate and analyzed with a H7600 transmission electron microscope (Hitachi).

### RNA isolation and microarray analysis

Exact timed matings were performed by overnight cohabitation of an inbred *Pax6^loxP/loxP^;DctCre* male with *Pax6^loxP/loxP^* females. Pregnant females were harvested on day E15.5 and the RPE of the embryos was dissected as previously described [18]. RPEs were pooled into two separate tubes according to their pigmentation intensity, and tubes were stored at −80°C. Tail cuts of the embryos were collected for genotype verification. Each tube was considered as one biological repeat. RNA was extracted using the QIAshredder and the RNeasy kits (QIAGEN). RNA isolated from three control and three mutant samples was processed for microarray analysis using the Affymetrix GeneChip 1.0ST as described previously [81]. Differentially expressed genes with p-values lower than 0.05 and with a fold-change cutoff of 1.5 are listed in Table S1. The expression data were submitted to the NCBI Gene Expression Omnibus (http://www.ncbi.nlm.nih.gov/geo) under series accession no. GSE56548.

### Reverse transcription and quantitative real-time and end-point PCR

Reverse transcription of 1 µg of RNA from each sample was performed using the SuperScript III First Strand kit (Invitrogen). cDNA was amplified using the Power SYBR Green Mix (Applied Biosystems) in a 384-well optical reaction plate using ABI Prism 7000 Sequence Detection System (Applied Biosystems). All primer pairs were first tested for specificity and amplicon size using end-point PCR. Formation of a dimer structure was refuted by analyzing the dissociation curve at the end of each amplification reaction. Results were calibrated in relation to an average of two house-keeping genes, *Ppia* and *Tbp*, after verifying that their levels were consistent in normal and mutant RPE. Raw data was processed using the comparative C_t_ method by the formula 2^−ΔΔCT^. Each amplification reaction was performed in triplicate using 20 ng of cDNA for each sample. Primers used to amplify and sequence the two *Pax6ΔPD* transcript variants are listed in Table S6.

### Luciferase reporter assay

Reporter assays were performed in HeLa cells using the Dual-Luciferase Reporter Assay System (Promega). Cells were seeded in a 24-well plate and 24 hours later were transfected using jetPEI DNA transfection reagent (Polyplus-transfection). Each well was co-transfected with three types of vectors in a total amount of 1210 ng of DNA: 1) 400 ng of a luciferase reporter vector (pGL3 basic) under the regulation of the examined promoter; 2) A total of 800 ng of expression vector (p3XFlag-CMV-10), either carrying no insert or containing an insert encoding the ORF of *Pax6*, *Pax6ΔPD* or *A-Mitf*, 400 ng of each; 3) 10 ng normalizing vector (pRL-TK). Cells were harvested 48 hours after transfection and luminescence was evaluated. Each treatment was carried out in duplicate, and each assay was repeated at least three times.

### Site-directed mutagenesis

End-point PCR of 17 cycles was performed using oligonucleotides containing the desired mutated sites (Table S6, mutated nucleotides are in lower case) and the wild-type promoter reporter plasmid (pGL3 basic) as template. The PCR products were treated with 12U *Dpn*I restriction enzyme (Fermentas) for 1 hour at 37°C, and 5 µl of the DNA was transformed into *E. coli XL-1Blue* strain, followed by colony-picking mini-prep and midi-prep plasmid purification (Qiagen). All plasmids were verified by sequencing.

### Immunoprecipitation and immunoblotting

Transfection into HeLa cells was performed as described in the reporter assay section. Cells were seeded in 90-mm dishes and transfected with total of 10 µg of DNA. Cells were washed with phosphate buffered saline (PBS), scraped in 1 ml lysis buffer (10 mM HEPES pH 8.0, 100 mM NaCl, 1 mM MgCl_2_, 0.5% NP-40) containing protease inhibitor (Roche, complete Mini EDTA-free) and incubated on ice for 30 minutes. Extracts were clarified by centrifugation at 10,000 g for 15 minutes at 4°C. To avoid nonspecific binding of proteins to the beads, extracts were subjected to pre-clearing using 15 µl of protein A agarose beads (Millipore, 16-157) for 2 hours at 4°C, followed by centrifugation at 10,000 g for 1 minute at 4°C. Input samples (50 µl of the supernatant) were kept at −20°C for input analysis and the cleared extracts were incubated with 5 µl of rabbit anti-MITF (kindly provided by David E. Fisher, MGH [82]) with rotation overnight at 4°C. The resulting immuno-complexes were incubated with 30 µl of protein A beads for 2 hours at 4°C. The beads were then washed four times with RIPA buffer (50 mM Tris-HCl pH 8.0, 150 mM NaCl, 1% NP-40, 0.5% Na-deoxycholate, 0.1% SDS) and the complexes were eluted in SDS sample buffer by boiling for 5 minutes. Samples were subjected to SDS–polyacrylamide gel electrophoresis. Separated proteins were transferred to nitrocellulose membrane and reacted with mouse anti-FLAG antibody (1∶10,000, Sigma F3165) followed by anti-mouse horseradish peroxidase-conjugated secondary antibody. The reaction was examined by enhanced chemiluminescence detection kit (Biological Industries).

Co-immunoprecipitation from ARPE19 cells was performed essentially as described above, except cells were scraped in RIPA buffer containing protease inhibitor (Roche, complete Mini EDTA-free). Antibodies used for IP were either rabbit anti-PAX6 (Millipore, AB2237) or rabbit anti-MIT (kindly provided by David E. Fisher, MGH). Mouse anti-PAX6 (Santa Cruz, sc-3276) and mouse anti-MITF (kindly provided by David E. Fisher, MGH [82]) antibodies were used for immunoblot.

ChIP was performed as previously described [83], [84]. Briefly, hES-RPE cells were grown as described [52]. Fixed chromatin was extracted from 2×10^7^ cells and immunoprecipitated using rabbit anti-PAX6 (Millipore, AB2237) or non-immune rabbit IgG (Rockland) as a negative control. The primers used for ChIP analysis are listed in Table S6.

### Electrophoretic mobility shift assay (EMSA)

HEK-293T cells were transfected with p3XFlag-CMV-10 encoding the ORF of full-length *Pax6*. Nuclear extracts were obtained as previously described [85]. Nuclear extract (1 ml) or 1∶10 diluted nuclear extract was incubated for 10 minutes on ice in 8.5 mM HEPES pH 7.9, 30 mM KCl, 1.5 mM MgCl_2_, 0.4 mM DTT, 2 mg polydI/dC (Sigma). Binding with 1 ml double-stranded 59-c-ATP-labeled probe (30,000 cpm) was performed at room temperature for 20 minutes and 200 ng of ‘‘cold’’ PAX6 consensus site (PAX6CON) was used for competition [86].

## Supporting Information

Figure S1 A PAX6ΔPD protein is expressed in the RPE of *Pax6^loxP/loxP^;DctCre* mutant mice. Paraffin sections of (A-D) control, *Pax6^loxP/loxP^*, and (E-H) mutant, *Pax6^loxP/loxP^;DctCre*, eyes stained for the N-terminus (red, amino acids 1-206) and C-terminus (green, last 18 amino acids) of PAX6. (A'-H' insets) Higher magnifications of indicated regions and nuclear staining with DAPI. (E-H') PAX6PD is not detected in the RPE of *Pax6^loxP/loxP^;DctCre* mutants at E12.5, E15.5 or E19.5 (red). Nevertheless, a PAX6ΔPD polypeptide is detected in the *Pax6^loxP/loxP^;DctCre* RPE (green). The spatiotemporal expression pattern of PAX6 isoforms is similar in wild-type and *Pax6^loxP/loxP^;DctCre* RPE: (A,E) at E12.5, PAX6 isoforms are expressed in the entire RPE; (B,F) at E15.5, PAX6 isoforms are highly expressed in the distal RPE; (D,H) and at E19.5 PAX6 isoforms are expressed only in the distal most cells of the RPE. (C',D',G',H' insets) The boundary region along the RPE, where the expression of PAX6 isoform is gradually reduced, is shown in higher magnifications. Scale bar is 50 µm.
(TIF)

Figure S2 ***Pax6*** **gene structure and transcripts expressed in the RPE of** ***Pax6^loxP/loxP^;DctCre*** **mutant and control mice.** (A) A scheme of *Pax6ΔPD* transcript expressed under the regulation of the P4 promoter. Striped rectangle indicates the location of the ISH probe used to identify the expression pattern of the *Pax6ΔPD* transcribed from promoter P4. (B) A scheme of the abnormal *Pax6ΔPD* transcript that is expressed in the RPE of *Pax6^loxP/loxP^;DctCre* mice. Coding exons are marked with large rectangles and non-coding exons are marked with small rectangles. The PD and HD coding exons are marked in light blue and blue, respectively. Locations of primers that were used to sequence the two *Pax6ΔPD* variants are marked with red arrows. Locations of possible start codons for the *Pax6ΔPD* transcript variants are indicated by ATG codons. (C) PCR products generated using primers designed to ampliy a *Pax6* intron 7 to exon 9 fragment, suggestive of the product shown in panel A. (D) PCR products generated using primers designed to ampliy the a *Pax6* exon 3 to exon 8 fragment, suggestive of the product shown in panel B. (E) A view of the distal OC of cryo-section subjected to *in situ* hybridization with a probe corresponding to *Pax6* intron 7. Scale bar is 50 µm. (F) Transcript levels of *Pax6* exons 7-8 in the RPE of control *Pax6^loxP/loxP^* and mutant *Pax6^loxP/loxP^;DctCre* mice at E15.5.
(TIF)

Figure S3 Expression of the RPE transcription factors *Otx2* and *Sox9* was similar in wild-type and *Pax6^loxP/loxP^;DctCre* mice. (A) Relative transcript levels of *Otx2* and *Sox9* in RPE fractions from wild-type and mutant mice determined using QRT-PCR (n = 5). (B-E) Distal OC view of paraffin sections labeled with antibodies against (B,D) OTX2 and (C,E) SOX9. Scale bar is 25 µm. Only few cells in the distal most region of the RPE of *Pax6^loxP/loxP^;DctCre* do not express OTX2 (B',D' insets). The expression of SOX9 in a proximal to distal gradient is maintained in the RPE of *Pax6^loxP/loxP^;DctCre* mutants. (C',E' insets) The boundary region along the RPE, where the expression of SOX9 gradually increases.
(TIF)

Figure S4 Schemes of the mouse *Mitf* gene locus, the deleted DNA segment in the *Mitf^ΔD/ΔD^* transgenic mice and the different MITF isoforms expressed in the RPE and choroid. (A) Scheme of *Mitf* gene structure presenting only the exons that constitute the four main isoforms expressed in the RPE (*A*-, *D*- and *H-Mitf*) and choroid (*M-Mitf*). The alternative transcription (TSS) and translation (ATG) start sites are indicated. White rectangles mark untranslated regions and colored rectangles mark coding sequences. A graphic scheme of all known *Mitf* isoforms and their unique alternative first exons is presented in Bharti et al. (2008) [18]. (B) The four MITF protein isoforms relevant to this study. Each isoform consist of different amino termini region. (C) An enlargement of the rectangular inset in (A). In the *Mitf^ΔD^* transgenic allele, a 5.8 kb DNA fragment that includes exon *D* (25bp) and covers a region of ∼200bp downstream and ∼5.6 kb upstream to the *D-Mitf* TSS was replaced by a neomycin cassette [37].
(TIF)

Figure S5 PAX6 trans-activates the promoters of *mMlana* in the presence of MITF. Activity of luciferase under the regulation of the *mMlana* promoter co-transfected into HeLa cells along with different combinations of expression vectors and/or their backbones lacking the ORF, as indicated (n = 3). The positions of binding sites for MITF (E-box, green rectangles) and potential binding sites for PAX6 (light blue rectangles) are indicated relative to the TSS.
(TIF)

Figure S6 The PAX6ΔPD variant is capable of association with MITF but probably does not take part in the MITF-mediated transcriptional activation of melanogenic genes. (A) co-IP of MITF and PAX6 or PAX6ΔPD. HeLa cells were transfected with vectors expressing 3xFlagPax6, 3xFlagMitf, 3xFlagPax6ΔPD or their combinations, as indicated. Cell lysates were prepared (input) and immunoprecipitated with anti-MITF antibodies. Samples were subjected to SDS-PAGE and analyzed by immunoblotting with anti-FLAG antibody (input: lanes 1-5; IP: lanes 6-10). Both full-length PAX6 and PAX6ΔPD were found to be in association with MITF (lanes 9-10). (B) Activity of luciferase under the regulation of wild-type m*Tyrp1* promoter co-transfected into HeLa cells along with different combinations and amounts of expression vectors and/or their backbones lacking the ORF, as indicated. PAX6ΔPD had no effect on PAX6-MITF mediated transactivation of the *mTyrp1* promoter.
(TIF)

Figure S7 The slight reduction in *Tfec* transcript level in the RPE of *Pax6^loxP/loxP^;DctCre* mice may contribute to the reduction in pigmentation. (A) Activity of luciferase under the regulation of wild-type m*Tyrp1* promoter (promoter scheme shown in Figure 5A) co-transfected into HeLa cells along with different combinations of expression vectors and/or their backbones lacking the ORF, as indicated. (B) Relative transcript levels of *Tfec* in wild-type and *Pax6^loxP/loxP^;DctCre* RPE fractions using QRT-PCR (n = 6).
(TIF)

Table S1 Genes differentially expressed in the *Pax6*-deficient RPE relative to wild-type RPE according to the microarray results.
(DOCX)

Tables S2 Putative MITF and PAX6 binding sites in *mD-Mitf* promoter (from +6 to −1153 relative to the TSS).
(DOCX)

Tables S3 Putative MITF and PAX6 binding sites in *mTyrp1* promoter (from +5 to −252 relative to the TSS).
(DOCX)

Tables S4 Putative MITF and PAX6 binding sites in *hTyr* promoter (from +80 to −115 relative to the TSS).
(DOCX)

Tables S5 Putative MITF and PAX6 binding sites in promoter of *mMlana* (from +6 to −1153 relative to the TSS).
(DOCX)

Table S6 Primers used in this study.
(DOCX)

Text S1 Supporting references.
(DOCX)

## References

[1] Strauss O (2005) The retinal pigment epithelium in visual function. Physiol Rev 85: 845–881. 15987797 10.1152/physrev.00021.2004

[2] del Marmol V, Beermann F (1996) Tyrosinase and related proteins in mammalian pigmentation. FEBS Lett 381: 165–168. 8601447 10.1016/0014-5793(96)00109-3

[3] Hearing VJ (1999) Biochemical control of melanogenesis and melanosomal organization. The journal of investigative dermatology Symposium proceedings 4: 24–28. 10.1038/sj.jidsp.5640176 10537003

[4] Burke JM (2008) Epithelial phenotype and the RPE: is the answer blowing in the Wnt? Prog Retin Eye Res 27: 579–595. 18775790 10.1016/j.preteyeres.2008.08.002 PMC2584165

[5] Davis N, Yoffe C, Raviv S, Antes R, Berger J, et al (2009) Pax6 Dosage Requirements in Iris and Ciliary Body Differentiation. Dev Biol 333: 132–42 doi: 10.1016/j.ydbio.2009.06.023 19563798 10.1016/j.ydbio.2009.06.023

[6] Fuhrmann S (2008) Wnt signaling in eye organogenesis. Organogenesis 4: 60–67. 19122781 10.4161/org.4.2.5850 PMC2613311

[7] Fuhrmann S, Levine EM, Reh TA (2000) Extraocular mesenchyme patterns the optic vesicle during early eye development in the embryonic chick. Development 127: 4599–4609. 11023863 10.1242/dev.127.21.4599

[8] Hyer J, Mima T, Mikawa T (1998) FGF1 patterns the optic vesicle by directing the placement of the neural retina domain. Development 125: 869–877. 9449669 10.1242/dev.125.5.869

[9] Mochii M, Mazaki Y, Mizuno N, Hayashi H, Eguchi G (1998) Role of Mitf in differentiation and transdifferentiation of chicken pigmented epithelial cell. Dev Biol 193: 47–62. 9466887 10.1006/dbio.1997.8800

[10] Nguyen M, Arnheiter H (2000) Signaling and transcriptional regulation in early mammalian eye development: a link between FGF and MITF. Development 127: 3581–3591. 10903182 10.1242/dev.127.16.3581

[11] Pittack C, Grunwald GB, Reh TA (1997) Fibroblast growth factors are necessary for neural retina but not pigmented epithelium differentiation in chick embryos. Development 124: 805–816. 9043062 10.1242/dev.124.4.805

[12] Westenskow P, Piccolo S, Fuhrmann S (2009) {beta}-catenin controls differentiation of the retinal pigment epithelium in the mouse optic cup by regulating Mitf and Otx2 expression. Development 136: 2505–2510. 19553286 10.1242/dev.032136 PMC2709060

[13] Zhao S, Overbeek PA (2001) Regulation of choroid development by the retinal pigment epithelium. Mol Vis 7: 277–282. 11740467

[14] Bharti K, Nguyen MT, Skuntz S, Bertuzzi S, Arnheiter H (2006) The other pigment cell: specification and development of the pigmented epithelium of the vertebrate eye. Pigment Cell Res 19: 380–394. 16965267 10.1111/j.1600-0749.2006.00318.x PMC1564434

[15] Aksan I, Goding CR (1998) Targeting the microphthalmia basic helix-loop-helix-leucine zipper transcription factor to a subset of E-box elements in vitro and in vivo. Mol Cell Biol 18: 6930–6938. 9819381 10.1128/mcb.18.12.6930 PMC109276

[16] Lowings P, Yavuzer U, Goding CR (1992) Positive and negative elements regulate a melanocyte-specific promoter. Mol Cell Biol 12: 3653–3662. 1321344 10.1128/mcb.12.8.3653-3662.1992 PMC364632

[17] Hemesath TJ, Steingrimsson E, McGill G, Hansen MJ, Vaught J, et al (1994) microphthalmia, a critical factor in melanocyte development, defines a discrete transcription factor family. Genes Dev 8: 2770–2780. 7958932 10.1101/gad.8.22.2770

[18] Bharti K, Liu W, Csermely T, Bertuzzi S, Arnheiter H (2008) Alternative promoter use in eye development: the complex role and regulation of the transcription factor MITF. Development 135: 1169–1178. 18272592 10.1242/dev.014142 PMC2276638

[19] Li XH, Kishore AH, Dao D, Zheng W, Roman CA, et al (2010) A novel isoform of microphthalmia-associated transcription factor inhibits IL-8 gene expression in human cervical stromal cells. Mol Endocrinol 24: 1512–1528. 20573688 10.1210/me.2009-0320 PMC2940468

[20] Hodgkinson CA, Moore KJ, Nakayama A, Steingrimsson E, Copeland NG, et al (1993) Mutations at the mouse microphthalmia locus are associated with defects in a gene encoding a novel basic-helix-loop-helix-zipper protein. Cell 74: 395–404. 8343963 10.1016/0092-8674(93)90429-t

[21] Levy C, Khaled M, Fisher DE (2006) MITF: master regulator of melanocyte development and melanoma oncogene. Trends Mol Med 12: 406–414. 16899407 10.1016/j.molmed.2006.07.008

[22] Takeda K, Yasumoto K, Kawaguchi N, Udono T, Watanabe K, et al (2002) Mitf-D, a newly identified isoform, expressed in the retinal pigment epithelium and monocyte-lineage cells affected by Mitf mutations. Biochim Biophys Acta 1574: 15–23. 11955610 10.1016/s0167-4781(01)00339-6

[23] Fuse N, Yasumoto K, Takeda K, Amae S, Yoshizawa M, et al (1999) Molecular cloning of cDNA encoding a novel microphthalmia-associated transcription factor isoform with a distinct amino-terminus. J Biochem 126: 1043–1051. 10578055 10.1093/oxfordjournals.jbchem.a022548

[24] Takemoto CM, Yoon YJ, Fisher DE (2002) The identification and functional characterization of a novel mast cell isoform of the microphthalmia-associated transcription factor. J Biol Chem 277: 30244–30252. 12039954 10.1074/jbc.M201441200

[25] Udono T, Yasumoto K, Takeda K, Amae S, Watanabe K, et al (2000) Structural organization of the human microphthalmia-associated transcription factor gene containing four alternative promoters. Biochim Biophys Acta 1491: 205–219. 10760582 10.1016/s0167-4781(00)00051-8

[26] Yajima I, Sato S, Kimura T, Yasumoto K, Shibahara S, et al (1999) An L1 element intronic insertion in the black-eyed white (Mitf[mi-bw]) gene: the loss of a single Mitf isoform responsible for the pigmentary defect and inner ear deafness. Hum Mol Genet 8: 1431–1441. 10400990 10.1093/hmg/8.8.1431

[27] Bondurand N, Pingault V, Goerich DE, Lemort N, Sock E, et al (2000) Interaction among SOX10, PAX3 and MITF, three genes altered in Waardenburg syndrome. Hum Mol Genet 9: 1907–1917. 10942418 10.1093/hmg/9.13.1907

[28] Potterf SB, Furumura M, Dunn KJ, Arnheiter H, Pavan WJ (2000) Transcription factor hierarchy in Waardenburg syndrome: regulation of MITF expression by SOX10 and PAX3. Hum Genet 107: 1–6. 10982026 10.1007/s004390000328

[29] Price ER, Horstmann MA, Wells AG, Weilbaecher KN, Takemoto CM, et al (1998) alpha-Melanocyte-stimulating hormone signaling regulates expression of microphthalmia, a gene deficient in Waardenburg syndrome. J Biol Chem 273: 33042–33047. 9830058 10.1074/jbc.273.49.33042

[30] Takeda K, Yasumoto K, Takada R, Takada S, Watanabe K, et al (2000) Induction of melanocyte-specific microphthalmia-associated transcription factor by Wnt-3a. J Biol Chem 275: 14013–14016. 10747853 10.1074/jbc.c000113200

[31] Watanabe A, Takeda K, Ploplis B, Tachibana M (1998) Epistatic relationship between Waardenburg syndrome genes MITF and PAX3. Nat Genet 18: 283–286. 9500554 10.1038/ng0398-283

[32] Baumer N, Marquardt T, Stoykova A, Spieler D, Treichel D, et al (2003) Retinal pigmented epithelium determination requires the redundant activities of Pax2 and Pax6. Development 130: 2903–2915. 12756174 10.1242/dev.00450

[33] Shaham O, Menuchin Y, Farhy C, Ashery-Padan R (2012) Pax6: a multi-level regulator of ocular development. Prog Retin Eye Res 31: 351–376. 22561546 10.1016/j.preteyeres.2012.04.002

[34] Kozmik Z (2005) Pax genes in eye development and evolution. Curr Opin Genet Dev 15: 430–438. 15950457 10.1016/j.gde.2005.05.001

[35] Chow RL, Altmann CR, Lang RA, Hemmati-Brivanlou A (1999) Pax6 induces ectopic eyes in a vertebrate. Development 126: 4213–4222. 10477290 10.1242/dev.126.19.4213

[36] Davidson EH, Erwin DH (2006) Gene regulatory networks and the evolution of animal body plans. Science 311: 796–800. 16469913 10.1126/science.1113832

[37] Bharti K, Gasper M, Ou J, Brucato M, Clore-Gronenborn K, et al (2012) A Regulatory Loop Involving PAX6, MITF, and WNT Signaling Controls Retinal Pigment Epithelium Development. PLoS Genet 8: e1002757. 22792072 10.1371/journal.pgen.1002757 PMC3390378

[38] Drager UC (1985) Birth dates of retinal ganglion cells giving rise to the crossed and uncrossed optic projections in the mouse. Proc R Soc Lond B Biol Sci 224: 57–77. 2581263 10.1098/rspb.1985.0021

[39] Strongin AC, Guillery RW (1981) The distribution of melanin in the developing optic cup and stalk and its relation to cellular degeneration. J Neurosci 1: 1193–1204. 7310485 10.1523/JNEUROSCI.01-11-01193.1981 PMC6564218

[40] Ashery-Padan R, Marquardt T, Zhou X, Gruss P (2000) Pax6 activity in the lens primordium is required for lens formation and for correct placement of a single retina in the eye. Genes Dev 14: 2701–2711. 11069887 10.1101/gad.184000 PMC317031

[41] Zhao S, Overbeek PA (1999) Tyrosinase-related protein 2 promoter targets transgene expression to ocular and neural crest-derived tissues. Dev Biol 216: 154–163. 10588869 10.1006/dbio.1999.9480

[42] Chen J, Bardes EE, Aronow BJ, Jegga AG (2009) ToppGene Suite for gene list enrichment analysis and candidate gene prioritization. Nucleic Acids Res 37: W305–311. 19465376 10.1093/nar/gkp427 PMC2703978

[43] Gibbs D, Azarian SM, Lillo C, Kitamoto J, Klomp AE, et al (2004) Role of myosin VIIa and Rab27a in the motility and localization of RPE melanosomes. J Cell Sci 117: 6473–6483. 15572405 10.1242/jcs.01580 PMC2942070

[44] Cheli Y, Ohanna M, Ballotti R, Bertolotto C (2010) Fifteen-year quest for microphthalmia-associated transcription factor target genes. Pigment Cell Melanoma Res 23: 27–40. 19995375 10.1111/j.1755-148X.2009.00653.x

[45] Steingrimsson E (2010) Interpretation of complex phenotypes: lessons from the Mitf gene. Pigment Cell Melanoma Res 23: 736–740. 21823251 10.1111/j.1755-148x.2010.00769.x

[46] Yasumoto K, Yokoyama K, Takahashi K, Tomita Y, Shibahara S (1997) Functional analysis of microphthalmia-associated transcription factor in pigment cell-specific transcription of the human tyrosinase family genes. J Biol Chem 272: 503–509. 8995290 10.1074/jbc.272.1.503

[47] Du J, Miller AJ, Widlund HR, Horstmann MA, Ramaswamy S, et al (2003) MLANA/MART1 and SILV/PMEL17/GP100 are transcriptionally regulated by MITF in melanocytes and melanoma. Am J Pathol 163: 333–343. 12819038 10.1016/S0002-9440(10)63657-7 PMC1868174

[48] Loftus SK, Antonellis A, Matera I, Renaud G, Baxter LL, et al (2009) Gpnmb is a melanoblast-expressed, MITF-dependent gene. Pigment Cell Melanoma Res 22: 99–110. 18983539 10.1111/j.1755-148X.2008.00518.x PMC2714741

[49] Chiaverini C, Beuret L, Flori E, Busca R, Abbe P, et al (2008) Microphthalmia-associated transcription factor regulates RAB27A gene expression and controls melanosome transport. J Biol Chem 283: 12635–12642. 18281284 10.1074/jbc.M800130200

[50] Vetrini F, Auricchio A, Du J, Angeletti B, Fisher DE, et al (2004) The microphthalmia transcription factor (Mitf) controls expression of the ocular albinism type 1 gene: link between melanin synthesis and melanosome biogenesis. Mol Cell Biol 24: 6550–6559. 15254223 10.1128/MCB.24.15.6550-6559.2004 PMC444869

[51] Galibert MD, Yavuzer U, Dexter TJ, Goding CR (1999) Pax3 and regulation of the melanocyte-specific tyrosinase-related protein-1 promoter. J Biol Chem 274: 26894–26900. 10480898 10.1074/jbc.274.38.26894

[52] Idelson M, Alper R, Obolensky A, Ben-Shushan E, Hemo I, et al (2009) Directed differentiation of human embryonic stem cells into functional retinal pigment epithelium cells. Cell Stem Cell 5: 396–408. 19796620 10.1016/j.stem.2009.07.002

[53] Vugler A, Carr AJ, Lawrence J, Chen LL, Burrell K, et al (2008) Elucidating the phenomenon of HESC-derived RPE: anatomy of cell genesis, expansion and retinal transplantation. Exp Neurol 214: 347–361. 18926821 10.1016/j.expneurol.2008.09.007

[54] Planque N, Leconte L, Coquelle FM, Martin P, Saule S (2001) Specific Pax-6/microphthalmia transcription factor interactions involve their DNA-binding domains and inhibit transcriptional properties of both proteins. J Biol Chem 276: 29330–29337. 11350962 10.1074/jbc.M101812200

[55] Perron M, Boy S, Amato MA, Viczian A, Koebernick K, et al (2003) A novel function for Hedgehog signalling in retinal pigment epithelium differentiation. Development 130: 1565–1577. 12620982 10.1242/dev.00391

[56] Holme RH, Thomson SJ, Davidson DR (2000) Ectopic expression of Msx2 in chick retinal pigmented epithelium cultures suggests a role in patterning the optic vesicle. Mech Dev 91: 175–187. 10704842 10.1016/s0925-4773(99)00296-8

[57] Fujimura N, Taketo MM, Mori M, Korinek V, Kozmik Z (2009) Spatial and temporal regulation of Wnt/beta-catenin signaling is essential for development of the retinal pigment epithelium. Dev Biol 334: 31–45. 19596317 10.1016/j.ydbio.2009.07.002

[58] Hallsson JH, Haflidadottir BS, Schepsky A, Arnheiter H, Steingrimsson E (2007) Evolutionary sequence comparison of the Mitf gene reveals novel conserved domains. Pigment Cell Res 20: 185–200. 17516926 10.1111/j.1600-0749.2007.00373.x

[59] Bovolenta P, Mallamaci A, Briata P, Corte G, Boncinelli E (1997) Implication of OTX2 in pigment epithelium determination and neural retina differentiation. J Neurosci 17: 4243–4252. 9151741 10.1523/JNEUROSCI.17-11-04243.1997 PMC6573571

[60] Martinez-Morales JR, Signore M, Acampora D, Simeone A, Bovolenta P (2001) Otx genes are required for tissue specification in the developing eye. Development 128: 2019–2030. 11493524 10.1242/dev.128.11.2019

[61] Martinez-Morales JR, Dolez V, Rodrigo I, Zaccarini R, Leconte L, et al (2003) OTX2 activates the molecular network underlying retina pigment epithelium differentiation. J Biol Chem 278: 21721–21731. 12663655 10.1074/jbc.M301708200

[62] Takeda K, Yokoyama S, Yasumoto K, Saito H, Udono T, et al (2003) OTX2 regulates expression of DOPAchrome tautomerase in human retinal pigment epithelium. Biochem Biophys Res Commun 300: 908–914. 12559959 10.1016/s0006-291x(02)02934-0

[63] Reinisalo M, Putula J, Mannermaa E, Urtti A, Honkakoski P (2012) Regulation of the human tyrosinase gene in retinal pigment epithelium cells: the significance of transcription factor orthodenticle homeobox 2 and its polymorphic binding site. Mol Vis 18: 38–54. 22259223 PMC3258524

[64] Hallsson JH, Haflidadottir BS, Stivers C, Odenwald W, Arnheiter H, et al (2004) The basic helix-loop-helix leucine zipper transcription factor Mitf is conserved in Drosophila and functions in eye development. Genetics 167: 233–241. 15166150 10.1534/genetics.167.1.233 PMC1470875

[65] Marquardt T, Ashery-Padan R, Andrejewski N, Scardigli R, Guillemot F, et al (2001) Pax6 is required for the multipotent state of retinal progenitor cells. Cell 105: 43–55. 11301001 10.1016/s0092-8674(01)00295-1

[66] Oron-Karni V, Farhy C, Elgart M, Marquardt T, Remizova L, et al (2008) Dual requirement for Pax6 in retinal progenitor cells. Development 135: 4037–4047. 19004853 10.1242/dev.028308 PMC4231183

[67] Shaham O, Smith AN, Robinson ML, Taketo MM, Lang RA, et al (2009) Pax6 is essential for lens fiber cell differentiation. Development 136: 2567–2578. 19570848 10.1242/dev.032888 PMC2709063

[68] Smith AN, Miller LA, Radice G, Ashery-Padan R, Lang RA (2009) Stage-dependent modes of Pax6-Sox2 epistasis regulate lens development and eye morphogenesis. Development 136: 2977–2985. 19666824 10.1242/dev.037341 PMC2723069

[69] Huang J, Rajagopal R, Liu Y, Dattilo LK, Shaham O, et al (2011) The mechanism of lens placode formation: a case of matrix-mediated morphogenesis. Dev Biol 355: 32–42. 21540023 10.1016/j.ydbio.2011.04.008 PMC3104088

[70] Grindley JC, Davidson DR, Hill RE (1995) The role of Pax-6 in eye and nasal development. Development 121: 1433–1442. 7789273 10.1242/dev.121.5.1433

[71] Kim J, Lauderdale JD (2006) Analysis of Pax6 expression using a BAC transgene reveals the presence of a paired-less isoform of Pax6 in the eye and olfactory bulb. Dev Biol 292: 486–505. 16464444 10.1016/j.ydbio.2005.12.041

[72] Kim J, Lauderdale JD (2008) Overexpression of pairedless Pax6 in the retina disrupts corneal development and affects lens cell survival. Dev Biol 313: 434–454. 18062951 10.1016/j.ydbio.2007.10.043

[73] Feuda R, Hamilton SC, McInerney JO, Pisani D (2012) Metazoan opsin evolution reveals a simple route to animal vision. Proc Natl Acad Sci U S A 109: 18868–18872. 23112152 10.1073/pnas.1204609109 PMC3503164

[74] Gehring WJ, Ikeo K (1999) Pax 6: mastering eye morphogenesis and eye evolution. Trends Genet 15: 371–377. 10461206 10.1016/s0168-9525(99)01776-x

[75] Suga H, Tschopp P, Graziussi DF, Stierwald M, Schmid V, et al (2010) Flexibly deployed Pax genes in eye development at the early evolution of animals demonstrated by studies on a hydrozoan jellyfish. Proc Natl Acad Sci U S A 107: 14263–14268. 20660753 10.1073/pnas.1008389107 PMC2922549

[76] Doughtie DG, Rao KR (1984) Ultrastructure of the eyes of the grass shrimp, Palaemonetes pugio. General morphology, and light and dark adaption at noon. Cell Tissue Res 238: 271–288.

[77] Davidson EH (2011) Evolutionary bioscience as regulatory systems biology. Dev Biol 357: 35–40. 21320483 10.1016/j.ydbio.2011.02.004 PMC3135751

[78] Yaron O, Farhy C, Marquardt T, Applebury M, Ashery-Padan R (2006) Notch1 functions to suppress cone-photoreceptor fate specification in the developing mouse retina. Development 133: 1367–1378. 16510501 10.1242/dev.02311

[79] Baxter LL, Pavan WJ (2003) Pmel17 expression is Mitf-dependent and reveals cranial melanoblast migration during murine development. Gene Expr Patterns 3: 703–707. 14643677 10.1016/j.modgep.2003.07.002

[80] Baba T, Bhutto IA, Merges C, Grebe R, Emmert D, et al (2010) A rat model for choroidal neovascularization using subretinal lipid hydroperoxide injection. Am J Pathol 176: 3085–3097. 20395434 10.2353/ajpath.2010.090989 PMC2877867

[81] Shaham O, Gueta K, Mor E, Oren-Giladi P, Grinberg D, et al (2013) Pax6 Regulates Gene Expression in the Vertebrate Lens through miR-204. PLoS Genet 9: e1003357. 23516376 10.1371/journal.pgen.1003357 PMC3597499

[82] Levy C, Khaled M, Robinson KC, Veguilla RA, Chen PH, et al (2010) Lineage-specific transcriptional regulation of DICER by MITF in melanocytes. Cell 141: 994–1005. 20550935 10.1016/j.cell.2010.05.004 PMC2897150

[83] Sailaja BS, Takizawa T, Meshorer E (2012) Chromatin immunoprecipitation in mouse hippocampal cells and tissues. Methods Mol Biol 809: 353–364. 22113288 10.1007/978-1-61779-376-9_24

[84] Sailaja BS, Cohen-Carmon D, Zimmerman G, Soreq H, Meshorer E (2012) Stress-induced epigenetic transcriptional memory of acetylcholinesterase by HDAC4. Proc Natl Acad Sci U S A 109: E3687–3695. 23236169 10.1073/pnas.1209990110 PMC3535662

[85] Hay-Koren A, Caspi M, Zilberberg A, Rosin-Arbesfeld R (2011) The EDD E3 ubiquitin ligase ubiquitinates and up-regulates beta-catenin. Mol Biol Cell 22: 399–411. 21118991 10.1091/mbc.E10-05-0440 PMC3031469

[86] Wolf LV, Yang Y, Wang J, Xie Q, Braunger B, et al (2009) Identification of pax6-dependent gene regulatory networks in the mouse lens. PLoS One 4: e4159. 19132093 10.1371/journal.pone.0004159 PMC2612750

[87] Hoashi T, Sato S, Yamaguchi Y, Passeron T, Tamaki K, et al (2010) Glycoprotein nonmetastatic melanoma protein b, a melanocytic cell marker, is a melanosome-specific and proteolytically released protein. FASEB J 24: 1616–29. 20056711 10.1096/fj.09-151019 PMC2879953

[88] Cortese K, Giordano F, Surace EM, Venturi C, Ballabio A, et al (2005) The ocular albinism type 1 (OA1) gene controls melanosome maturation and size. Invest Ophthalmol Vis Sci 46: 4358–4364. 16303920 10.1167/iovs.05-0834

[89] Incerti B, Cortese K, Pizzigoni A, Surace EM, Varani S, et al (2000) Oa1 knock-out: new insights on the pathogenesis of ocular albinism type 1. Hum Mol Genet 9: 2781–2788. 11092754 10.1093/hmg/9.19.2781

[90] Costin GE, Valencia JC, Vieira WD, Lamoreux ML, Hearing VJ (2003) Tyrosinase processing and intracellular trafficking is disrupted in mouse primary melanocytes carrying the underwhite (uw) mutation. A model for oculocutaneous albinism (OCA) type 4. J Cell Sci 116: 3203–3212. 12829739 10.1242/jcs.00598

[91] Du J, Fisher DE (2002) Identification of Aim-1 as the underwhite mouse mutant and its transcriptional regulation by MITF. J Biol Chem 277: 402–406. 11700328 10.1074/jbc.M110229200

[92] Wu XS, Rao K, Zhang H, Wang F, Sellers JR, et al (2002) Identification of an organelle receptor for myosin-Va. Nat Cell Biol 4: 271–278. 11887186 10.1038/ncb760

[93] Vogel P, Read RW, Vance RB, Platt KA, Troughton K, et al (2008) Ocular albinism and hypopigmentation defects in Slc24a5-/- mice. Vet Pathol 45: 264–279. 18424845 10.1354/vp.45-2-264

[94] Dooley TP, Curto EV, Davis RL, Grammatico P, Robinson ES, et al (2003) DNA microarrays and likelihood ratio bioinformatic methods: discovery of human melanocyte biomarkers. Pigment Cell Res 16: 245–253. 12753397 10.1034/j.1600-0749.2003.00036.x

[95] Baxter LL, Pavan WJ (2002) The oculocutaneous albinism type IV gene Matp is a new marker of pigment cell precursors during mouse embryonic development. Mech Dev 116: 209–212. 12128226 10.1016/s0925-4773(02)00130-2

