# Supplementary figures and images for: PAX6 Regulates Melanogenesis in the Retinal Pigmented Epithelium through Feed-Forward Regulatory Interactions with MITF
Source: PLoS Genet. 2014 May 29;10(5):e1004360. doi: 10.1371/journal.pgen.1004360 (PMC4038462; doi:10.1371/journal.pgen.1004360)

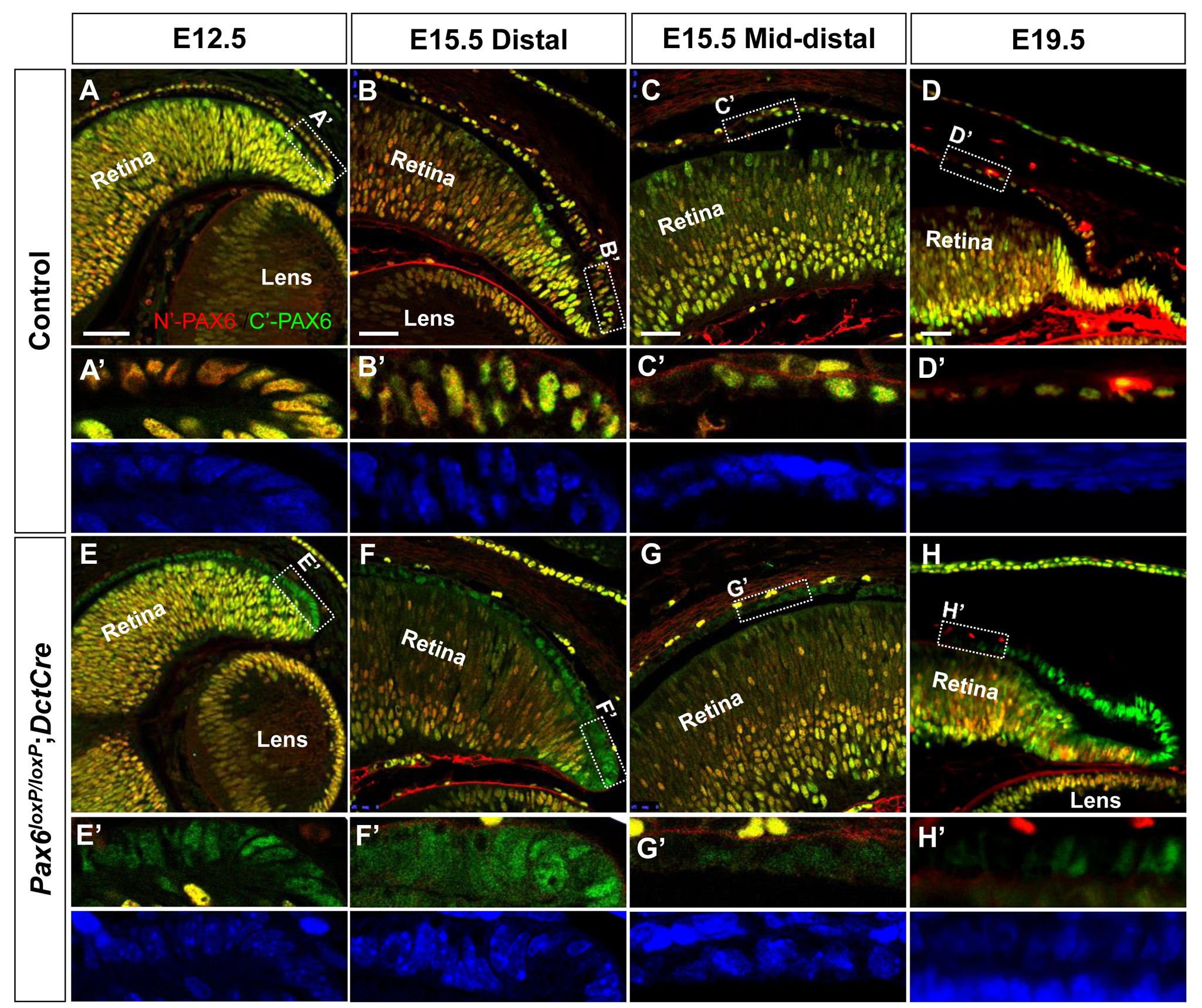

Supplement: Figure S1 — A PAX6ΔPD protein is expressed in the RPE of Pax6loxP/loxP;DctCre mutant mice. Paraffin sections of (A-D) control, Pax6loxP/loxP, and (E-H) mutant, Pax6loxP/loxP;DctCre, eyes stained for the N-terminus (red, amino acids 1-206) and C-terminus (green, last 18 amino acids) of PAX6. (A'-H' insets) Higher magnifications of indicated regions and nuclear staining with DAPI. (E-H') PAX6PD is not detected in the RPE of Pax6loxP/loxP;DctCre mutants at E12.5, E15.5 or E19.5 (red). Nevertheless, a PAX6ΔPD polypeptide is detected in the Pax6loxP/loxP;DctCre RPE (green). The spatiotemporal expression pattern of PAX6 isoforms is similar in wild-type and Pax6loxP/loxP;DctCre RPE: (A,E) at E12.5, PAX6 isoforms are expressed in the entire RPE; (B,F) at E15.5, PAX6 isoforms are highly expressed in the distal RPE; (D,H) and at E19.5 PAX6 isoforms are expressed only in the distal most cells of the RPE. (C',D',G',H' insets) The boundary region along the RPE, where the expression of PAX6 isoform is gradually reduced, is shown in higher magnifications. Scale bar is 50 µm. (TIF) [file pgen.1004360.s001.tif]

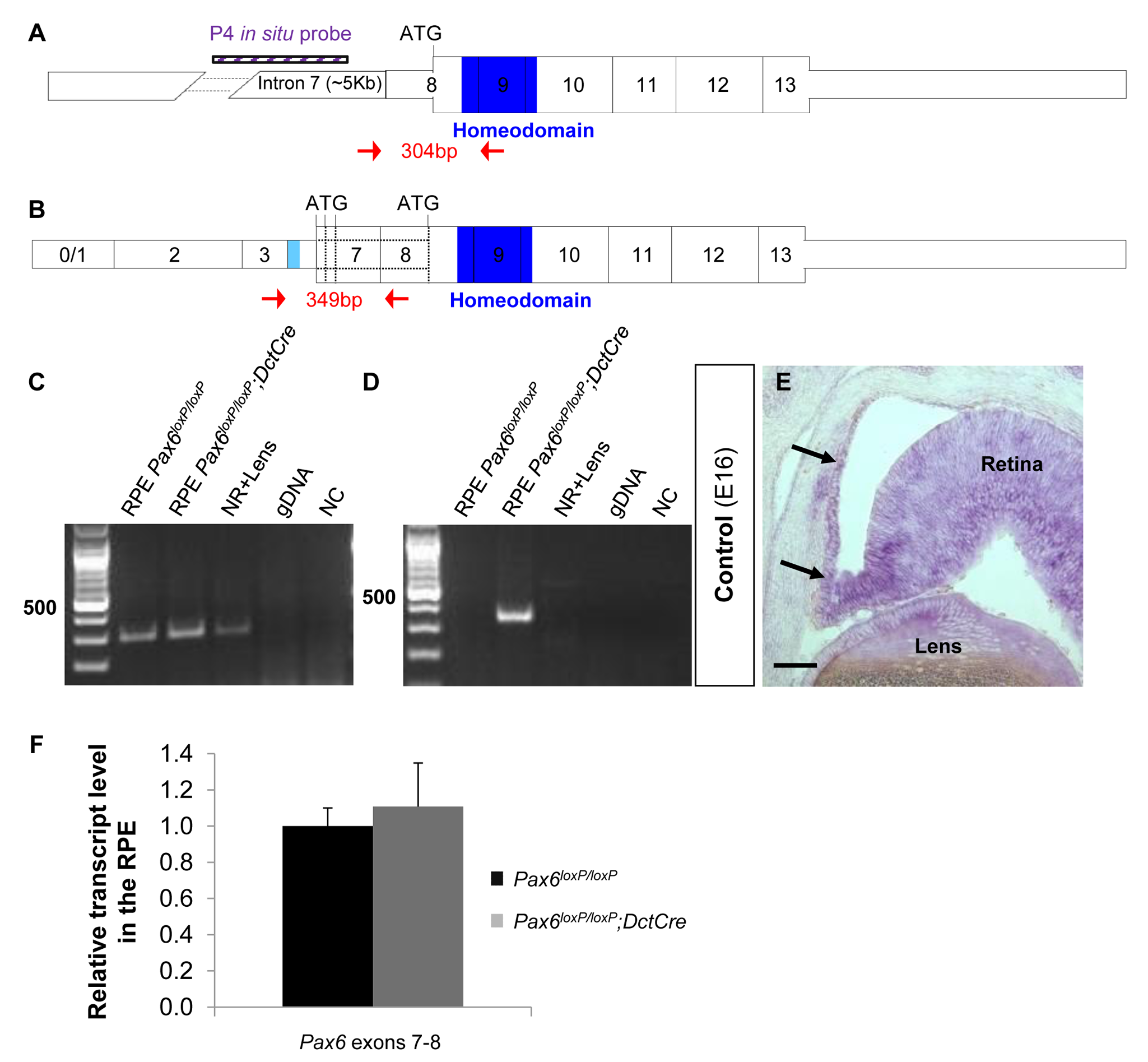

Supplement: Figure S2 — Pax6 gene structure and transcripts expressed in the RPE of Pax6loxP/loxP;DctCre mutant and control mice. (A) A scheme of Pax6ΔPD transcript expressed under the regulation of the P4 promoter. Striped rectangle indicates the location of the ISH probe used to identify the expression pattern of the Pax6ΔPD transcribed from promoter P4. (B) A scheme of the abnormal Pax6ΔPD transcript that is expressed in the RPE of Pax6loxP/loxP;DctCre mice. Coding exons are marked with large rectangles and non-coding exons are marked with small rectangles. The PD and HD coding exons are marked in light blue and blue, respectively. Locations of primers that were used to sequence the two Pax6ΔPD variants are marked with red arrows. Locations of possible start codons for the Pax6ΔPD transcript variants are indicated by ATG codons. (C) PCR products generated using primers designed to ampliy a Pax6 intron 7 to exon 9 fragment, suggestive of the product shown in panel A. (D) PCR products generated using primers designed to ampliy the a Pax6 exon 3 to exon 8 fragment, suggestive of the product shown in panel B. (E) A view of the distal OC of cryo-section subjected to in situ hybridization with a probe corresponding to Pax6 intron 7. Scale bar is 50 µm. (F) Transcript levels of Pax6 exons 7-8 in the RPE of control Pax6loxP/loxP and mutant Pax6loxP/loxP;DctCre mice at E15.5. (TIF) [file pgen.1004360.s002.tif]

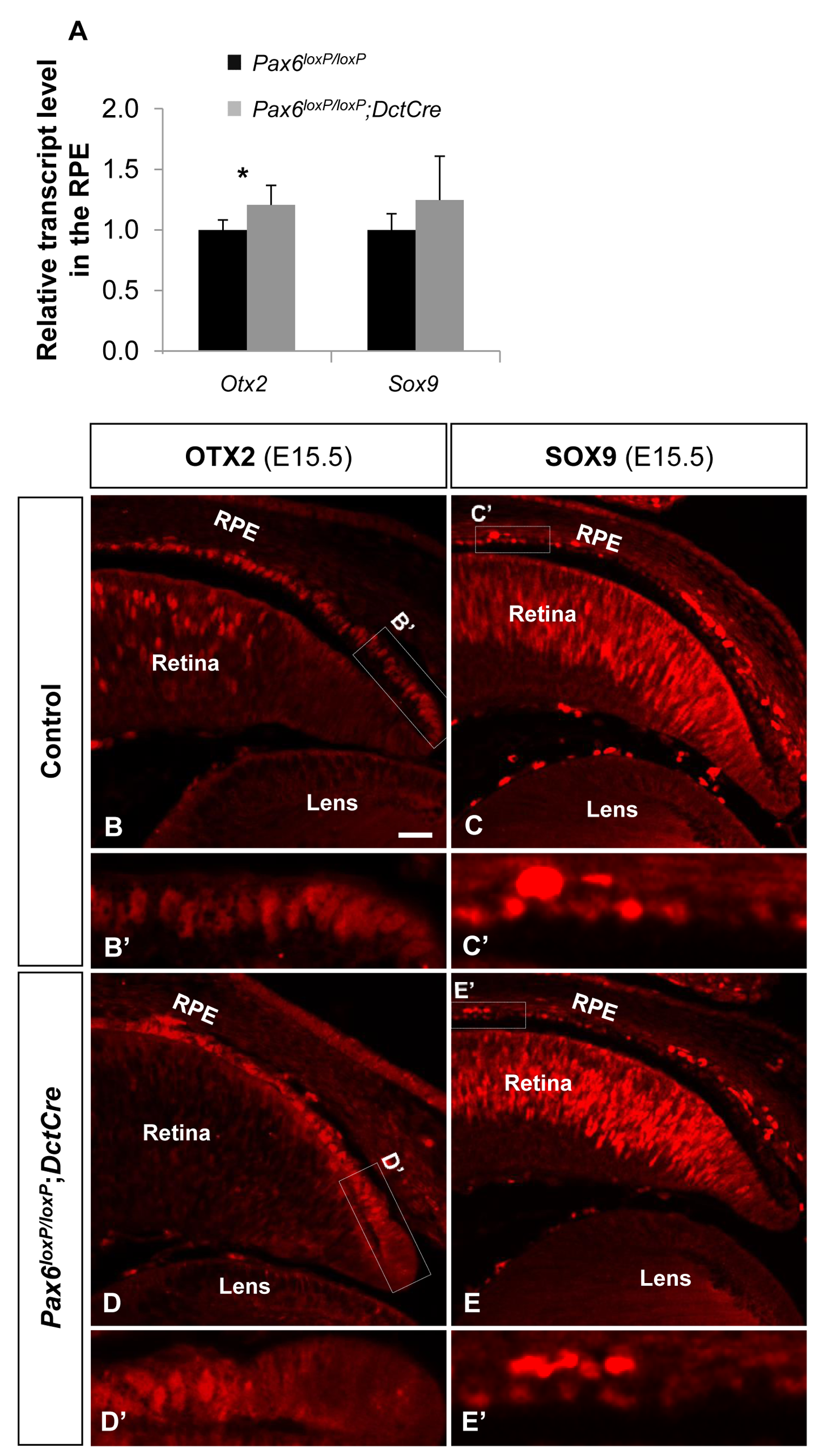

Supplement: Figure S3 — Expression of the RPE transcription factors Otx2 and Sox9 was similar in wild-type and Pax6loxP/loxP;DctCre mice. (A) Relative transcript levels of Otx2 and Sox9 in RPE fractions from wild-type and mutant mice determined using QRT-PCR (n = 5). (B-E) Distal OC view of paraffin sections labeled with antibodies against (B,D) OTX2 and (C,E) SOX9. Scale bar is 25 µm. Only few cells in the distal most region of the RPE of Pax6loxP/loxP;DctCre do not express OTX2 (B',D' insets). The expression of SOX9 in a proximal to distal gradient is maintained in the RPE of Pax6loxP/loxP;DctCre mutants. (C',E' insets) The boundary region along the RPE, where the expression of SOX9 gradually increases. (TIF) [file pgen.1004360.s003.tif]

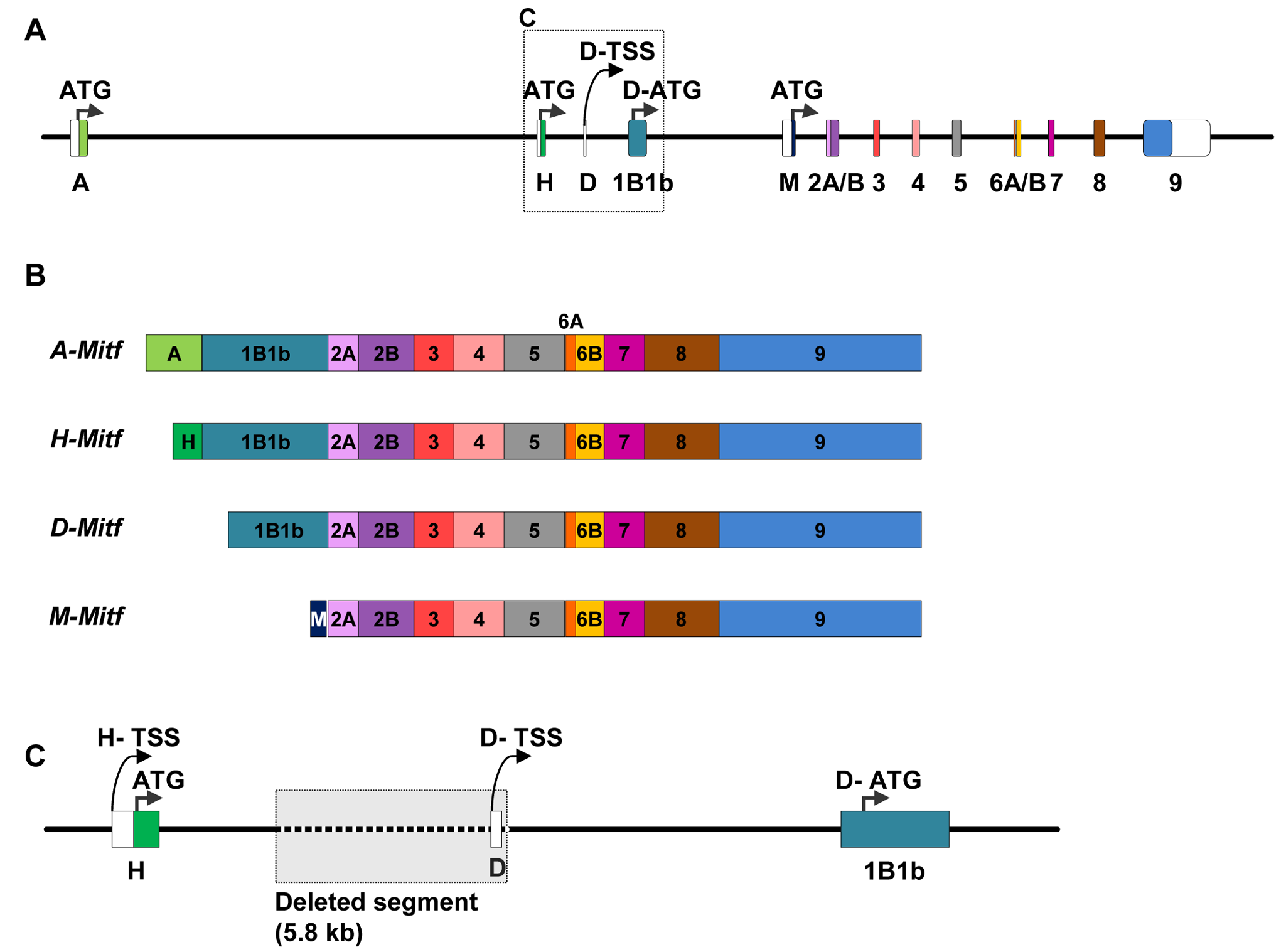

Supplement: Figure S4 — Schemes of the mouse Mitf gene locus, the deleted DNA segment in the MitfΔD/ΔD transgenic mice and the different MITF isoforms expressed in the RPE and choroid. (A) Scheme of Mitf gene structure presenting only the exons that constitute the four main isoforms expressed in the RPE (A-, D- and H-Mitf) and choroid (M-Mitf). The alternative transcription (TSS) and translation (ATG) start sites are indicated. White rectangles mark untranslated regions and colored rectangles mark coding sequences. A graphic scheme of all known Mitf isoforms and their unique alternative first exons is presented in Bharti et al. (2008) [18]. (B) The four MITF protein isoforms relevant to this study. Each isoform consist of different amino termini region. (C) An enlargement of the rectangular inset in (A). In the MitfΔD transgenic allele, a 5.8 kb DNA fragment that includes exon D (25bp) and covers a region of ∼200bp downstream and ∼5.6 kb upstream to the D-Mitf TSS was replaced by a neomycin cassette [37]. (TIF) [file pgen.1004360.s004.tif]

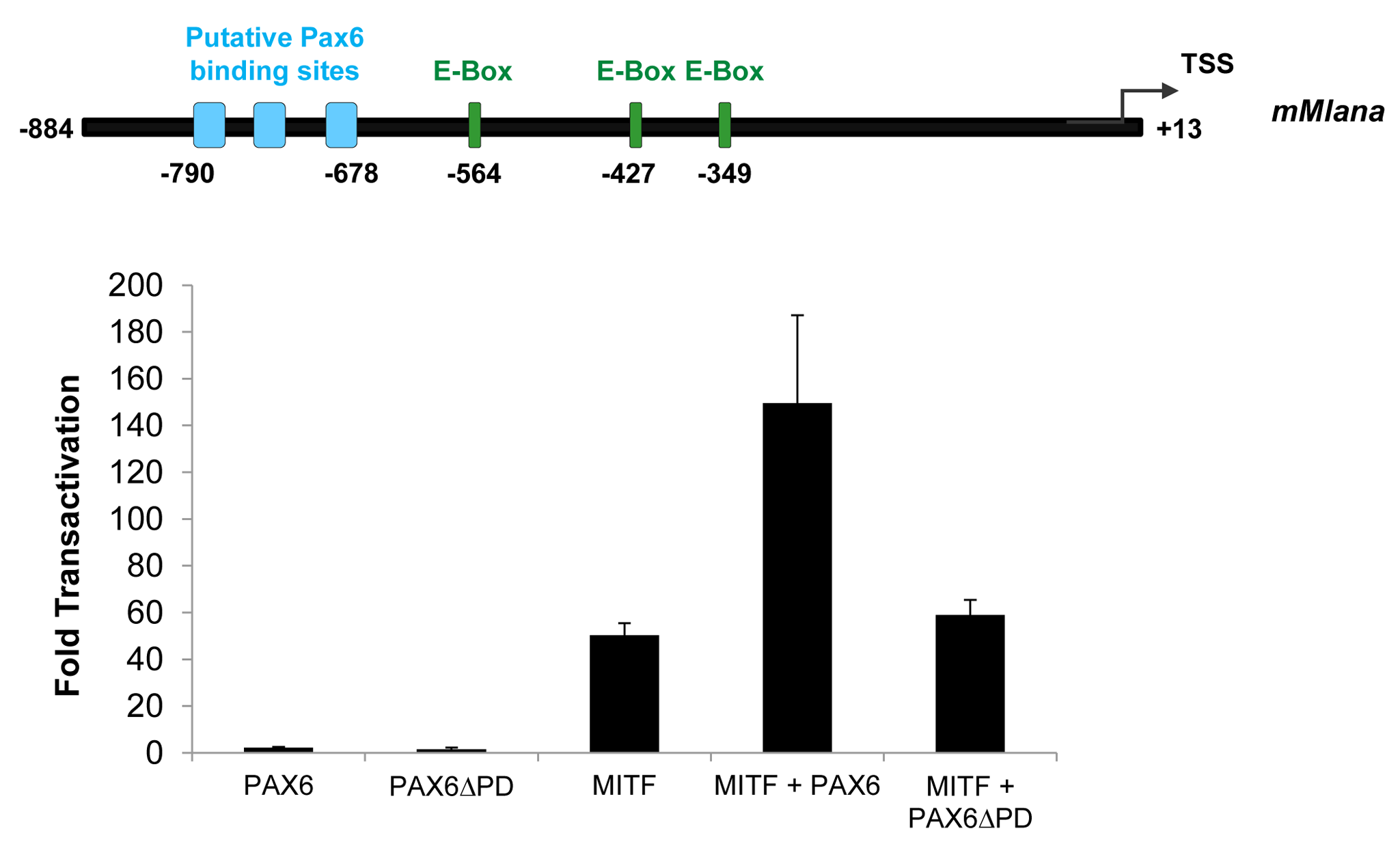

Supplement: Figure S5 — PAX6 trans-activates the promoters of mMlana in the presence of MITF. Activity of luciferase under the regulation of the mMlana promoter co-transfected into HeLa cells along with different combinations of expression vectors and/or their backbones lacking the ORF, as indicated (n = 3). The positions of binding sites for MITF (E-box, green rectangles) and potential binding sites for PAX6 (light blue rectangles) are indicated relative to the TSS. (TIF) [file pgen.1004360.s005.tif]

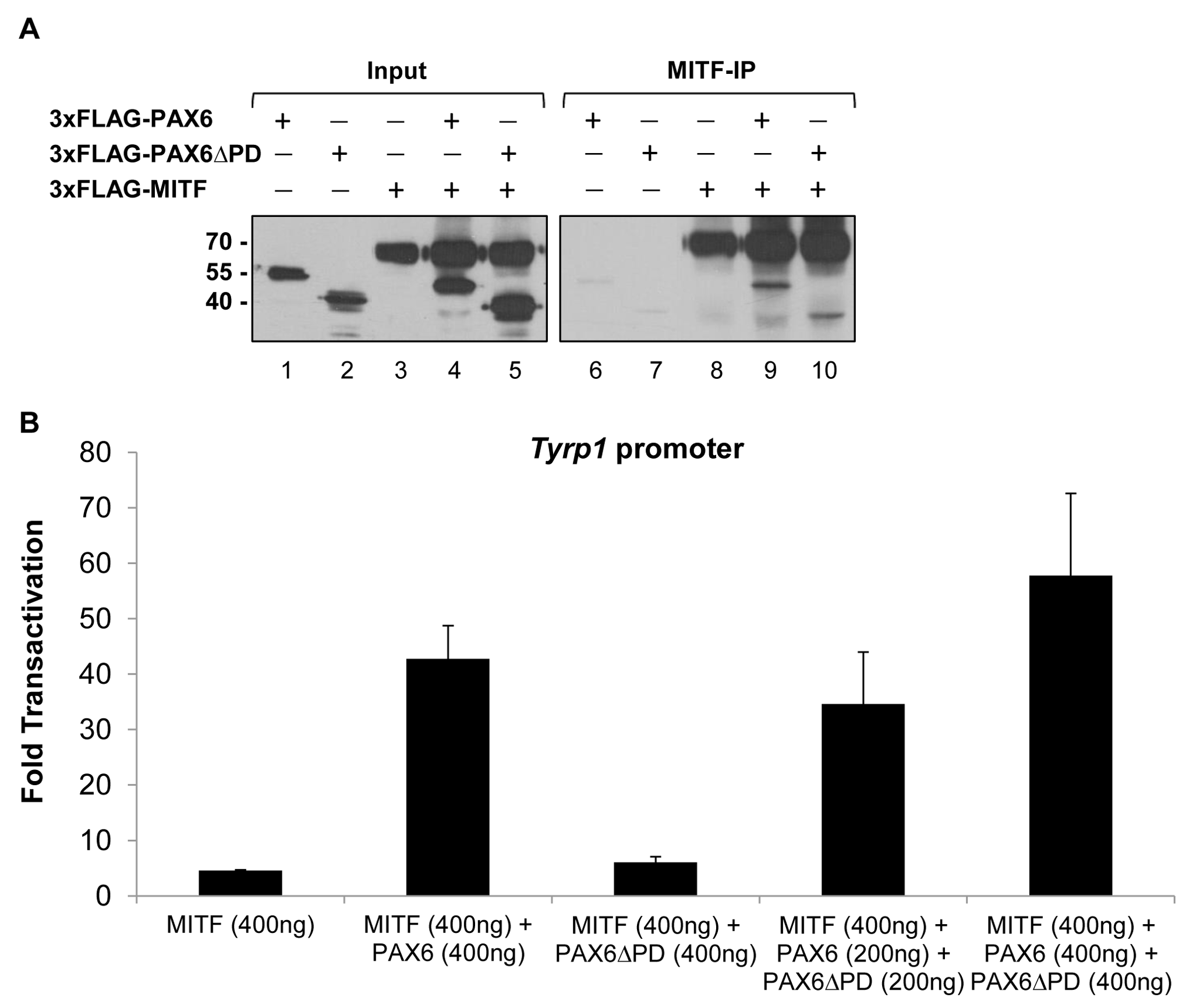

Supplement: Figure S6 — The PAX6ΔPD variant is capable of association with MITF but probably does not take part in the MITF-mediated transcriptional activation of melanogenic genes. (A) co-IP of MITF and PAX6 or PAX6ΔPD. HeLa cells were transfected with vectors expressing 3xFlagPax6, 3xFlagMitf, 3xFlagPax6ΔPD or their combinations, as indicated. Cell lysates were prepared (input) and immunoprecipitated with anti-MITF antibodies. Samples were subjected to SDS-PAGE and analyzed by immunoblotting with anti-FLAG antibody (input: lanes 1-5; IP: lanes 6-10). Both full-length PAX6 and PAX6ΔPD were found to be in association with MITF (lanes 9-10). (B) Activity of luciferase under the regulation of wild-type mTyrp1 promoter co-transfected into HeLa cells along with different combinations and amounts of expression vectors and/or their backbones lacking the ORF, as indicated. PAX6ΔPD had no effect on PAX6-MITF mediated transactivation of the mTyrp1 promoter. (TIF) [file pgen.1004360.s006.tif]

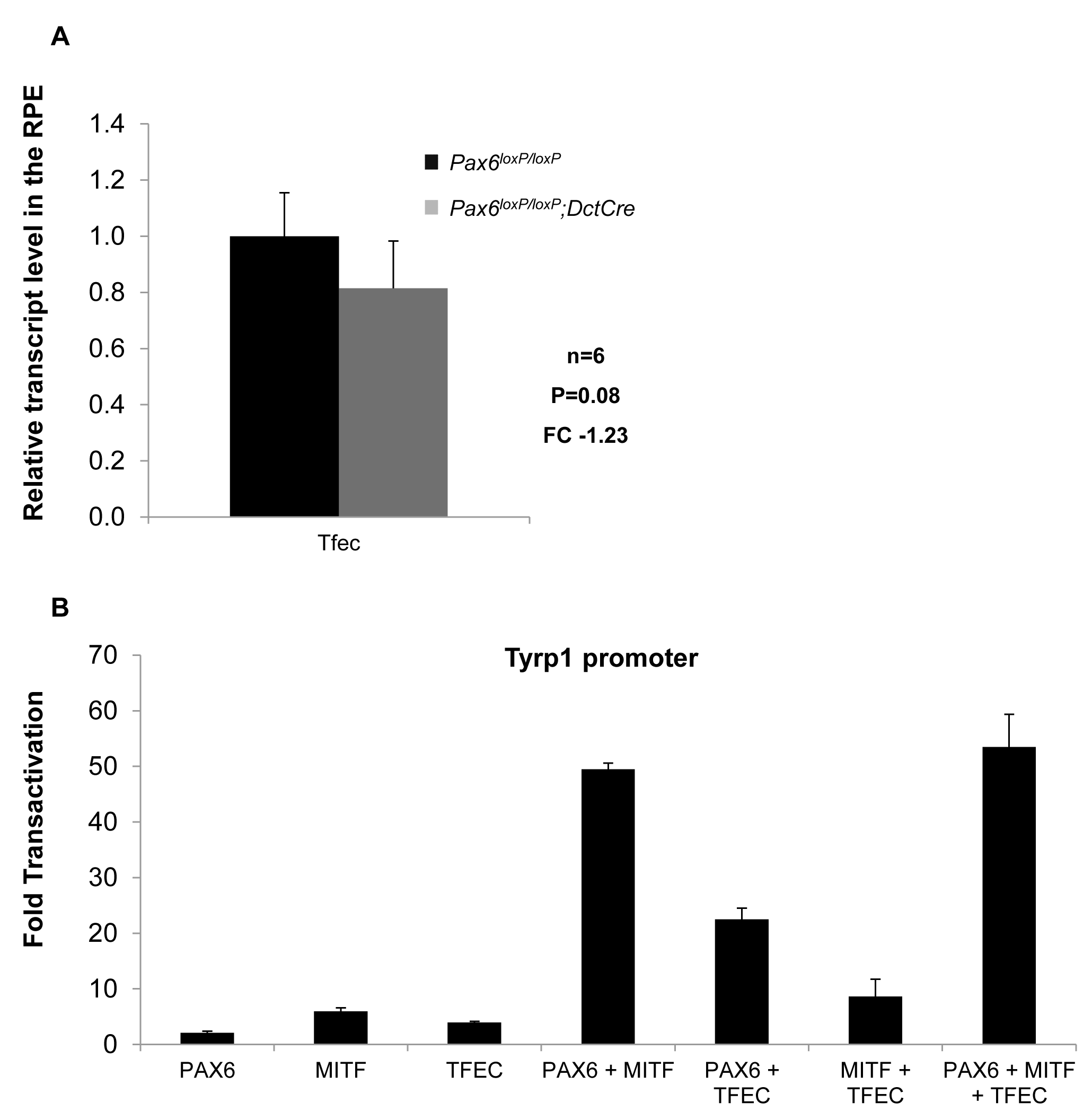

Supplement: Figure S7 — The slight reduction in Tfec transcript level in the RPE of Pax6loxP/loxP;DctCre mice may contribute to the reduction in pigmentation. (A) Activity of luciferase under the regulation of wild-type mTyrp1 promoter (promoter scheme shown in Figure 5A) co-transfected into HeLa cells along with different combinations of expression vectors and/or their backbones lacking the ORF, as indicated. (B) Relative transcript levels of Tfec in wild-type and Pax6loxP/loxP;DctCre RPE fractions using QRT-PCR (n = 6). (TIF) [file pgen.1004360.s007.tif]
