# Supplementary material for: PAX6 Regulates Melanogenesis in the Retinal Pigmented Epithelium through Feed-Forward Regulatory Interactions with MITF
Source: PLoS Genet. 2014 May 29;10(5):e1004360. doi: 10.1371/journal.pgen.1004360 (PMC4038462; doi:10.1371/journal.pgen.1004360)
Supplement: Table S1 — Genes differentially expressed in the Pax6-deficient RPE relative to wild-type RPE according to the microarray results. (DOCX) [file pgen.1004360.s008.docx]

| **Gene symbol** | **Fold change** | **P-value** |
| --- | --- | --- |
| *Ttr* | 4.30 | 0.00026 |
| *Rgs9* | 2.63 | 0.00091 |
| *Tmem196* | 2.24 | 0.01330 |
| *Tbc1d9* | 2.20 | 0.00034 |
| *Car12* | 2.00 | 0.00212 |
| *Slco1a4* | 1.94 | 0.02757 |
| *Chrnb4* | 1.87 | 0.01396 |
| *Nov* | 1.84 | 0.00361 |
| *Msx2* | 1.81 | 0.01632 |
| *Hey2* | 1.80 | 0.00406 |
| *Accn3* | 1.79 | 0.01807 |
| *Wt1* | 1.78 | 0.00451 |
| *Cldn19* | 1.78 | 0.00678 |
| *Olfm3* | 1.74 | 0.01664 |
| *Frmd3* | 1.70 | 0.01209 |
| *Bmp6* | 1.67 | 0.00380 |
| *Mab21l2* | 1.66 | 0.02883 |
| *Zic1* | 1.63 | 0.03010 |
| *Rtn4rl1* | 1.62 | 0.00526 |
| *Rasef* | 1.61 | 0.00851 |
| *Enpp2* | 1.60 | 0.02369 |
| *Nrp2* | 1.60 | 0.02198 |
| *Lrrn3* | 1.59 | 0.00467 |
| *Atp13a4* | 1.55 | 0.00240 |
| *Htr2c* | 1.55 | 0.00603 |
| *Tbx20* | 1.51 | 0.00112 |
| *C130060K24Rik* | 1.50 | 0.00268 |
| *Folh1* | -1.51 | 0.00990 |
| *Kcnab1* | -1.52 | 0.00146 |
| *Lpl* | -1.52 | 0.00033 |
| *Gsta1* | -1.53 | 0.01374 |
| *Optn* | -1.53 | 0.00323 |
| *Slc16a12* | -1.54 | 0.00698 |
| *Krtdap* | -1.55 | 0.02404 |
| *Tst* | -1.56 | 0.00201 |
| *Tacr3* | -1.57 | 0.00726 |
| *Ugcg* | -1.57 | 0.00047 |
| *Bhlhe41* | -1.58 | 0.00840 |
| *Tyr* | -1.58 | 0.04166 |
| *Dsg3* | -1.60 | 0.00332 |
| *Si* | -1.60 | 0.00721 |
| *Cyp1b1* | -1.60 | 0.03184 |
| *Dpep1* | -1.62 | 0.01707 |
| *BC018465* | -1.62 | 0.00082 |
| *Gabrb3* | -1.63 | 0.00118 |
| *Stxbp5l* | -1.65 | 0.00007 |
| *Bdh2* | -1.65 | 0.01540 |
| *Slc3a2* | -1.66 | 0.00025 |
| *Wnt2b* | -1.67 | 0.00005 |
| *Zdhhc2* | -1.67 | 0.01002 |
| *Slc24a5* | -1.68 | 0.03025 |
| *Bnc1* | -1.68 | 0.00830 |
| *Gmpr* | -1.69 | 0.00072 |
| *Aldh3b2* | -1.70 | 0.02168 |
| *Ephx2* | -1.72 | 0.00199 |
| *St3gal6* | -1.72 | 0.00356 |
| *Tbc1d30* | -1.74 | 0.01639 |
| *Rbm11* | -1.76 | 0.00140 |
| *Elovl7* | -1.78 | 0.00054 |
| *Raet1b* | -1.79 | 0.00007 |
| *Chrm4* | -1.80 | 0.00082 |
| *Rab27a* | -1.81 | 0.00530 |
| *Folr1* | -1.81 | 0.00099 |
| *S100a1* | -1.82 | 0.00097 |
| *Uap1l1* | -1.83 | 0.00979 |
| *Guca1b* | -1.84 | 0.00008 |
| *Serpinb3a* | -1.85 | 0.01603 |
| *Tspan10* | -1.85 | 0.00330 |
| *Ifit3* | -1.88 | 0.00178 |
| *Rab3b* | -1.90 | 0.00002 |
| *Slc45a2* | -1.90 | 0.00630 |
| *Tyrp1* | -1.91 | 0.00297 |
| *Tmem27* | -1.93 | 0.00183 |
| *Bhlhe41* | -1.95 | 0.00519 |
| *Bace2* | -1.95 | 0.00046 |
| *Mgll* | -1.96 | 0.00057 |
| *Rlbp1* | -1.99 | 0.00173 |
| *I830012O16Rik* | -2.07 | 0.00043 |
| *Krt12* | -2.12 | 0.01137 |
| *Krt6a* | -2.16 | 0.00850 |
| *Kcnq5* | -2.24 | 0.00018 |
| *Dpp4* | -2.25 | 0.00005 |
| *Mapk4* | -2.35 | 0.00004 |
| *Nqo1* | -2.35 | 0.00042 |
| *C2* | -2.40 | 0.00030 |
| *Aldh3a1* | -2.49 | 0.00419 |
| *Rrh* | -2.53 | 0.00060 |
| *Slc7a8* | -2.61 | 0.00143 |
| *Clcnka* | -2.74 | 0.00001 |
| *Gpr143* | -2.83 | 0.00267 |
| *Slc4a5* | -2.90 | 0.00135 |
| *Myh11* | -3.41 | 0.00001 |
| *Opn4* | -3.46 | 0.00062 |
| *Slc11a1* | -3.52 | 0.00005 |
| *Ggt1* | -3.89 | 0.00006 |
| *Mlana* | -4.17 | 0.00018 |
| *Gpnmb* | -5.73 | 0.00020 |
| *Slc7a11* | -5.93 | 0.00002 |
| *Pcp4* | -7.03 | 0.00008 |
| *Slc38a8* | -16.90 | 0.00051 |
