# Supplementary material for: PAX6 Regulates Melanogenesis in the Retinal Pigmented Epithelium through Feed-Forward Regulatory Interactions with MITF
Source: PLoS Genet. 2014 May 29;10(5):e1004360. doi: 10.1371/journal.pgen.1004360 (PMC4038462; doi:10.1371/journal.pgen.1004360)
Supplement: Tables S2 — Putative MITF and PAX6 binding sites in mD-Mitf promoter (from +6 to −1153 relative to the TSS). (DOCX) [file pgen.1004360.s009.docx]

| **Binding site** | **Sequence** | **Coordinates relative to the TSS** | **Reference** |
| --- | --- | --- | --- |
| PAX6 PD | GGTTGAAGCATGAAGCCTT | -212 to -194 | Genomatix MatInspector algorithm [[1](#_ENREF_96)] |
| MITF (consensus E-box) | CATGTG | -315 to -310 | [[2](#_ENREF_46)] |
| MITF (E-box) | CATATG | -345 to -340 | [[3](#_ENREF_49),[4](#_ENREF_97)] |
| MITF (E-box) | CAGCTG | -382 to -377 | [[3](#_ENREF_49)] |
| MITF (consensus E-box) | CATGTG | -762 to -757 | [[2](#_ENREF_46)] |
| MITF (E-box) | CATCTG | -980 to -975 | [[5](#_ENREF_98)] |
| PAX6 PD | CATGAGGCATCTGGGAAGA | -987 to -969 | Genomatix MatInspector algorithm [[1](#_ENREF_96)] |
| PAX6 PD | GGTCAGGGAGGTGTCAGCA | -1105 to -1087 | Genomatix MatInspector algorithm [[1](#_ENREF_96)] |
