# Supplementary material for: PAX6 Regulates Melanogenesis in the Retinal Pigmented Epithelium through Feed-Forward Regulatory Interactions with MITF
Source: PLoS Genet. 2014 May 29;10(5):e1004360. doi: 10.1371/journal.pgen.1004360 (PMC4038462; doi:10.1371/journal.pgen.1004360)
Supplement: Tables S3 — Putative MITF and PAX6 binding sites in mTyrp1 promoter (from +5 to −252 relative to the TSS). (DOCX) [file pgen.1004360.s010.docx]

| **Binding site** | **Sequence** | **Coordinates relative to the TSS** | **Reference** |
| --- | --- | --- | --- |
| PAX6 | GAGAAGGGATTAGTGAGAG | -19 to -1 | Genomatix MatInspector algorithm [[1](#_ENREF_96)] |
| MITF (M-box) | AGTCATGTGCT | -66 to -56 | [[2](#_ENREF_46)] |
