# Supplementary material for: PAX6 Regulates Melanogenesis in the Retinal Pigmented Epithelium through Feed-Forward Regulatory Interactions with MITF
Source: PLoS Genet. 2014 May 29;10(5):e1004360. doi: 10.1371/journal.pgen.1004360 (PMC4038462; doi:10.1371/journal.pgen.1004360)
Supplement: Tables S4 — Putative MITF and PAX6 binding sites in hTyr promoter (from +80 to −115 relative to the TSS). (DOCX) [file pgen.1004360.s011.docx]

| **Binding site** | **Sequence** | **Coordinates relative to the TSS** | **Reference** |
| --- | --- | --- | --- |
| MITF (consensus E-box) | CATGTG | -9 to -4 | [[2](#_ENREF_46)] |
| PAX6 PD | ATTATATAGGTCTCAG | -31 to -16 | [6] |
| PAX6 PD | TGATGCTGGAG | -53 to -43 | [[6](#_ENREF_99)] |
| PAX6 PD | TAAAGACTAAAA | -70 to -59 | [[6](#_ENREF_99)] |
| MITF (M-box) | AGTCATGTGCT | -104 to -94 | [[2](#_ENREF_46)] |
