# Supplementary material for: PAX6 Regulates Melanogenesis in the Retinal Pigmented Epithelium through Feed-Forward Regulatory Interactions with MITF
Source: PLoS Genet. 2014 May 29;10(5):e1004360. doi: 10.1371/journal.pgen.1004360 (PMC4038462; doi:10.1371/journal.pgen.1004360)
Supplement: Tables S5 — Putative MITF and PAX6 binding sites in promoter of mMlana (from +6 to −1153 relative to the TSS). (DOCX) [file pgen.1004360.s012.docx]

| **Binding site** | **Sequence** | **Coordinates relative to the TSS** | **Reference** |
| --- | --- | --- | --- |
| MITF (consensus E-box) | CACATG | -345 to -340 | [[2](#_ENREF_46)] |
| MITF (consensus E-box) | CATGTG | -437 to -432 | [[2](#_ENREF_46)] |
| MITF (E-box) | CAACTG | -567 to -562 |  |
| PAX6 PD | CCTGTCCTGGAATGTGGTC | -696 to -678 | Genomatix MatInspector algorithm [[1](#_ENREF_96)] |
| PAX6 PD | CCTTCACTCCTGAAGTACA | -748 to -730 | Genomatix MatInspector algorithm [[1](#_ENREF_96)] |
| PAX6 PD | CCTCCTCACTTTCCTCCTC | -790 to -772 | Genomatix MatInspector algorithm [[1](#_ENREF_96)] |
