# Supplementary material for: PAX6 Regulates Melanogenesis in the Retinal Pigmented Epithelium through Feed-Forward Regulatory Interactions with MITF
Source: PLoS Genet. 2014 May 29;10(5):e1004360. doi: 10.1371/journal.pgen.1004360 (PMC4038462; doi:10.1371/journal.pgen.1004360)
Supplement: Table S6 — Primers used in this study. (DOCX) [file pgen.1004360.s013.docx]

| **A. Quantitative real-time PCR primers** | |
| --- | --- |
| **Amplicon** | **Sequence 5' – 3'** |
| *Ppia* (sense) | GCAAATGCTGGACCAAACAC |
| *Ppia* (anti sense) | TCACCTTCCCAAAGACCACAT |
| *Tbp* (sense) | CTTCGTGCAAGAAATGCTGAAT |
| *Tbp* (anti sense) | CAGTTGTCCGTGGCTCTCTTATT |
| *Connexin-43* (sense) | TTTGGCGTGCCGGCTTCACTTTC |
| *Connexin-43* (anti sense) | CCATGTCTGGGCACCTCTCTTTC |
| *P-cadherin* (sense) | GCGTCTCCTTCGAGCTGTTTG |
| *P-cadherin* (anti sense) | TCCAGTGCTCAGAGCTCGAAAG |
| *ZO-1* (sense) | TCCACAGCAGCCCAGCAATGG |
| *ZO-1* (anti sense) | CCCAGGTTTAGACATTCGCTCTTCC |
| *Pax6* (exons 7-8) (sense) | GACTTCAGTACCAGGGCAACC |
| *Pax6* (exons 7-8) | CTTTGCAGCTTCCGCTTCAG |
| pan *Mitf* (sense) | CCCGTCTCTGGAAACTTGATCGACC |
| pan *Mitf* (anti sense) | GTCGTTTATGTTAAATCTTCTTCTTCG |
| *M-Mitf* (sense) | GGAAATGCTAGAATACAGTCACTACC |
| *M-Mitf* (anti sense) | CATGCACGACGCTCGAGAGTGC |
| *A-Mitf* (sense) | GGCGGATTTCGAAGTCGGGGAGG |
| *D-Mitf* (sense) | GTTGGGACCTGACAGGCTCTGAATACAG |
| *H-Mitf* (sense) | TGGAGGCGCTTAGATTTGAGATGC |
| *A*-, *D*- and *H-Mitf* (anti sense) | GCACGCTGACGTTTATGGCTGG |
| *Tyr* (sense) | CTCAGCCCAGCATCCTTCTTCTC |
| *Tyr* (anti sense) | AGTGGTCCCTCAGGTGTTCCATC |
| *Tyrp1* (sense) | AGCTCTGTGCTGTATTTTCATCTGA |
| *Tyrp1* (anti sense) | GGTCTCCTATGCTGCTCTTTGC |
| *Si* (sense) | TATGGGTGTCCAGAGAAGGAGCTTC |
| *Si* (anti sense) | ACACCAAGCCAGTCCTGATTCCTG |
| *Mlana* (sense) | GAAATCCCATCAGCCCGTGGTTC |
| *Mlana* (anti sense) | GAGGGAAGTTGTTCAGCGTTCTCAG |
| *Dct* (sense) | GCTGATTAGTCGGAACTCGAGATTC |
| *Dct* (anti sense) | CATAGGTTCCATTACACAGTGTGACC |
| *Myo7a* (sense) | ACGCACGATGACTGGCAGATTG |
| *Myo7a* (anti sense) | AGCAACATGTCATGGTGGTCCTTC |
| *Otx2* (sense) | TCATGAGGGAAGAGGTGGCACTG |
| *Otx2* (anti sense) | TTGGCGGCACTTAGCTCTTCGATTC |
| *Sox9* (sense) | GCGAGCACTCTGGGCAATC |
| *Sox9* (anti sense) | CCCTCTCGCTTCAGATCAACTTTG |
| **B. Primers used for amplification of the in situ probe for Pax6 intron 7** | |
| *Pax6* (Intron 7) (sense) | TTTGGAGCCCTCCATCTTTCTC |
| *Pax6* (Intron 7) (anti sense) | TGCACCTTTCGGGCAAGG |
| **C. Primers used for sequencing the Pax6∆PD variants** | |
| *Pax6* (intron 7-exon 9) (sense) | CAGATTACCCAGTCCTCGGAGTTTC |
| *Pax6* (intron 7-exon 9) (anti sense) | CTTTCCCGGGCAAACACATCTG |
| *Pax6* (exons 3-8) (sense) | TTTAACCAAGGGCGGTGAGC |
| *Pax6* (exons 3-8) (anti sense) | CTTTGCAGCTTCCGCTTCAG |
| **D. Primers used for site-directed mutagenesis. Mutated nucleotides marked in lower case (sense primers were used with their corresponding antisense primers)** | |
| *Tyrp1* M-box (sense) | AGTGGGGAGGGAGTgAgGTGCTGCCTAGTAG |
| *Tyr* M-box (sense) | AAAGAAAAGTCAGTgAgGTGCTTTTCAGAGG |
| *Tyr* E-box (sense) | GTCTCAGCCAAGAgAgGTGATAATCACTG |
| **E. Primers used for ChIP analysis** | |
| *Tyrp1* enriched amplicon (sense) | CAGTTGGAAGGGAATCATGTG |
| *Tyrp1* enriched amplicon (anti sense) | ATGTGGATTGCTGCCTGATAA |
| *Tyrp1* negative control (sense) | GGTCGTGGTGTGTTACAAGATG |
| *Tyrp1* negative control (anti sense) | AGTTGTGCGCTTTGCCATAT |
