# Supplementary material for: PAX6 Regulates Melanogenesis in the Retinal Pigmented Epithelium through Feed-Forward Regulatory Interactions with MITF
Source: PLoS Genet. 2014 May 29;10(5):e1004360. doi: 10.1371/journal.pgen.1004360 (PMC4038462; doi:10.1371/journal.pgen.1004360)
Supplement: Text S1 — Supporting references. (DOCX) [file pgen.1004360.s014.docx]

**Supporting references**

1. Cartharius K, Frech K, Grote K, Klocke B, Haltmeier M, et al. (2005) MatInspector and beyond: promoter analysis based on transcription factor binding sites. Bioinformatics 21: 2933-2942.

2. Yasumoto K, Yokoyama K, Takahashi K, Tomita Y, Shibahara S (1997) Functional analysis of microphthalmia-associated transcription factor in pigment cell-specific transcription of the human tyrosinase family genes. J Biol Chem 272: 503-509.

3. Chiaverini C, Beuret L, Flori E, Busca R, Abbe P, et al. (2008) Microphthalmia-associated transcription factor regulates RAB27A gene expression and controls melanosome transport. J Biol Chem 283: 12635-12642.

4. Ge Y, Jippo T, Lee YM, Adachi S, Kitamura Y (2001) Independent influence of strain difference and mi transcription factor on the expression of mouse mast cell chymases. Am J Pathol 158: 281-292.

5. Morii E, Tsujimura T, Jippo T, Hashimoto K, Takebayashi K, et al. (1996) Regulation of mouse mast cell protease 6 gene expression by transcription factor encoded by the mi locus. Blood 88: 2488-2494.

6. Xie Q, Cvekl A (2011) The orchestration of mammalian tissue morphogenesis through a series of coherent feed-forward loops. J Biol Chem 286: 43259-43271.
